# Virtual reality programs targeting executive functions and social cognition evaluation and/or rehabilitation in children with ADHD or ASD—A narrative review

**DOI:** 10.3389/fpsyg.2025.1583052

**Published:** 2025-11-05

**Authors:** Filippia Doulou, Pascale Piolino, Nathalie Angeard

**Affiliations:** Laboratoire Mémoire, Cerveau et Cognition (UR 7536), Institut de Psychologie, Université de Paris Cité, Paris, France

**Keywords:** ADHD, ASD, assessment, neurodevelopmental disorders, pediatric population, training, virtual reality

## Abstract

Various studies have underlined the possible effectiveness of innovative techniques, such as virtual reality (VR), during the assessment or the rehabilitation of cognition in clinical pediatric populations. This study aims to (a) review the VR environments designed to assess and/or enhance executive functions (EFs) and theory of mind (ToM) domains in children and adolescents with neurodevelopmental disorders and (b) evaluate the sensitivity and the efficacy of these VR tools. Following an overview of these studies (e.g., purpose and results), our study has two further goals: (1) to provide the methodological dimensions of each study (target skills/processes and clinical populations), and (2) to highlight the VR characteristics (e.g., sense of presence and immersive experience, the user's point of view) implemented in the selected articles. A total of 75 studies published between 1996 and 2022 and fulfilling the selected criteria were found on database platforms such as PubMed or Science Direct. Our review demonstrates that VR could be useful as an assessment and training tool for cognitive and social impairments in pediatric clinical populations. However, the numerous clinical and VR designs highlight the need to develop a more systematic evaluation of VR programs to define what really works, especially in terms of generalization to more naturalistic settings.

## Highlights

- This study reviews 75 articles investigating the use of virtual reality (VR) for the assessment or training of cognitive [e.g., executive functions (EFs)] or social (e.g., emotion recognition) skills in children with neurodevelopmental disorders.
- The majority of the studies involving children with attention-deficit/hyperactivity disorders (ADHD) focused on the assessment and training of attentional impairments, whereas interventions targeting social skills predominantly involved autism spectrum disorder (ASD) participants.
- A high variability was found across studies in both clinical design (number and duration of training sessions) and virtual reality (VR) program characteristics, including device, user perspective (first person vs. third person), level of immersion, and interactivity.

## 1 Introduction

Neurodevelopmental disorders, characterized by an inability to reach cognitive, emotional, and motor developmental milestones, are typically linked to disruptions in the highly coordinated processes underlying brain development (Parenti et al., 2020; Thapar et al., 2017). Attention-deficit/hyperactivity disorders (ADHD), autism spectrum disorder (ASD), learning disabilities, and intellectual disability are emblematic examples of neurodevelopmental disorders. Executive function (EF) and theory of mind (ToM) impairments are considered two of the core cognitive dysfunctions commonly observed in individuals with autism spectrum disorder (ASD) and attention-deficit/hyperactivity disorder (ADHD) (Bora and Pantelis, 2016; Peterson and Wellman, 2019).

Executive functions (EFs) play a central role in the conscious regulation of thought and action (Pellicano, 2012) and are considered essential for cognitive development. In everyday life, individuals frequently encounter situations that require them to suppress heuristics in favor of more deliberate strategies such as reasoning or planning (Baddeley and Hitch, 1974; Norman and Shallice, 1986). Neurocognitive evidence supports an integrative theoretical model of EFs that incorporates domain-general systems (e.g., Central Executive Network and Salience Network) and underscores the dynamic interplay between automatic and controlled processing (Friedman and Robbins, 2022) throughout development (Diamond, 2013).

Recent studies have emphasized the developmental trajectory of executive functions (EFs) from infancy through late adolescence (Traverso et al., 2015), highlighting both their early emergence and gradual structural refinement. Between the ages of 3 and 8 years, a unidimensional EF structure differentiates into three core components: inhibition, cognitive flexibility, and working memory (Lee et al., 2013). Neurodevelopmental findings indicate a shift from diffuse to increasingly focal brain activation patterns, particularly within the prefrontal cortex, reflecting progressive modularization (Karmiloff-Smith, 2018) and functional specialization of brain regions associated with distinct EF components (Fiske and Holmboe, 2019). These findings align with the gradient of modularity in EF-related processing based on system complexity (demanding functional specialization) and expertise throughout learning (Benso et al., 2025). The protracted development and maturation of EF-related neural networks contribute to a heightened period of vulnerability during childhood. However, this extended maturation also implies significant neuroplasticity during sensitive developmental windows (Anderson et al., 2011), supporting the potential for effective intervention and training of EF skills (Kloo and Perner, 2008).

A growing body of research provides empirical evidence that executive dysfunction is a core characteristic of children with neurodevelopmental disorders such as autism spectrum disorder (ASD) and attention-deficit/hyperactivity disorder (ADHD). In ASD, deficits in executive functions have been consistently documented (Ciesielski and Harris, 1997; Robinson et al., 2009), while in ADHD, executive impairments are widely recognized and well established (Barkley and Murphy, 2010; Castellanos et al., 2006; Gualtieri and Johnson, 2005). Functional neuroimaging studies further support these findings by demonstrating associations between executive dysfunction and abnormal prefrontal cortex activity (O'Hearn et al., 2008), as well as disruptions in frontal–subcortical networks (Minzenberg et al., 2009; Niendam et al., 2012). Impairments in EFs may also contribute to difficulties in interpreting social situations and generating appropriate responses, which are frequently observed in both ASD and ADHD populations (Müller and Kerns, 2015; Pellicano, 2007, 2012; Pugliese et al., 2015; Tseng and Gau, 2013).

EFs are crucial for acquiring and understanding social rules, thereby serving as a foundation for the emergence or expression of social behavior. A closely related construct is social cognition, which is typically divided into four core domains: emotional processing, social perception, attributional style/bias, and theory of mind (ToM) (Fernández-Sotos et al., 2020). ToM refers to the ability to attribute mental states—such as beliefs, intentions, or emotions—to oneself and others, recognizing that these may differ across individuals (Hahs, 2015). ToM is considered a bidimensional construct that spans a continuum from affective to cognitive components (Canty et al., 2017b; Shamay-Tsoory et al., 2006). Cognitive ToM involves the ability to infer about others' beliefs and intentions, whereas affective ToM refers to understanding others' emotions by interpreting emotional or motivational cues within a given context (Canty et al., 2017a).

ToM develops gradually and follows a predictable sequence, as children progressively manage to understand and master complex mental states. Children first understand that individuals can have/express different desires or beliefs about the same situation. This precedes the capacity to grasp false beliefs—recognizing the distinction between one's own knowledge of reality and another person's incorrect belief (Wellman and Liu, 2004). Neuroimaging studies have identified a network of brain regions consistently associated with ToM processing, including the medial prefrontal cortex (mPFC), the posterior superior temporal sulcus (pSTS), the precuneus, the amygdala/temporopolar cortex, and the right temporoparietal junction (TPJ) (Gallagher and Frith, 2003; Peterson and Wellman, 2019).

The concept of ToM has been extensively explored in ASD research for over 35 years. It was first introduced by Baron-Cohen et al. (1985) through the false belief paradigm, who demonstrated that individuals with ASD experience difficulties in ToM-related tasks (Fletcher-Watson et al., 2014). Given the link between ToM and social-communication skills, many interventions targeting individuals with ASD aimed to enhance ToM and its precursor skills, including joint attention, imitation, and emotion recognition (Fletcher-Watson et al., 2014; Garfield et al., 2001).

Considering the hierarchical organization of complex domain-general EF systems and domain-specific systems, such as theory of mind, some researchers argue that ToM initially depends on EFs to emerge but gradually becomes autonomous (the emergence account) (Devine and Hughes, 2014). In contrast, others hold that ToM continues to rely on EFs across the lifespan, consistent with the expression account (Carlson et al., 2015; Devine and Hughes, 2014). Given the functional and cognitive plasticity of EFs during the preschool period as well as their crucial role in both social and cognitive development (Anderson et al., 2011; Dennis et al., 2014), several studies have investigated the effects of EF training in the general pediatric population (see Diamond and Ling, 2020, for a review). Moreover, a number of interventions have been developed to target ToM specifically in children with neurodevelopmental disorders (Fernández-Sotos et al., 2020; Fletcher-Watson et al., 2014). One example is the “thought-bubble” paradigm in which characters' mental states (e.g., thoughts and/or beliefs) are illustrated with cartoon-like bubbles (as in Rajendran, 1999).

## 2 Assessment and training in executive functions and/or theory of mind: Does virtual reality have a role to play?

### 2.1 The challenge of using classical laboratory settings to assess or train sociocognitive skills

Previous research has highlighted limitations in the standardized neuropsychological assessment of EFs, as mainly widely used tasks are multi-component and therefore fail to evaluate only a specific component. The lack of ecological validity is also considered a major drawback as it could limit the transfer of training to daily life (Anderson, 2002; Krasny-Pacini et al., 2016). Moreover, studies have emphasized that the discrepancy between traditional, non-immersive cognitive tasks and the complexity of real-life situations reduces the ecological validity and effectiveness of classical assessment tools (Loomis et al., 1999). Accordingly, a major limitation of EF or ToM interventions is the failure to generalize improved skills to contexts beyond the specific training protocol (Fletcher-Watson et al., 2014; Jolles and Crone, 2012). For example, Winner and Crooke (2014) argues that understanding the mental states of real people is a far more demanding task for ASD patients than interpreting the mental states of fictional characters in structured stories. Hofmann et al. (2016) pointed out that classical ToM trainings based on social scenarios fail to elicit meaningful motivational engagement from participants. This lack of engagement is attributed to two key factors: participants' difficulty/inability in shifting perspectives (from someone who experiences a situation to someone who merely witnesses a situation) (Frith and Frith, 2006) and participants' passivity/passive role (as the participant remains passive, mainly during the whole procedure). According to Parsons and Mitchell (2002), VR has the potential to facilitate the transfer of social skills from virtual to real-world contexts. Hence, VR can provide a safe, controlled, and immersive setting in which individuals can engage in role-play scenarios, thereby supporting the development of social problem-solving abilities. It could therefore be a highly promising tool for both the assessment of sociocognitive skills and interventions. However, despite the widespread use of the term “virtual reality,” current VR systems vary considerably in terms of technology, interactivity, and immersion levels, which poses challenges for standardization and cross-study comparisons.

### 2.2 VR: definition, advantages, and classification of VR environments

VR, also known as computer-simulated reality or video-generated environments, is a computer technology that simulates an imagined or real-like environment (Bashiri et al., 2017), such as a *café* (Mitchell et al., 2007) or a classroom (Rizzo et al., 2000). By using this technology, users can interact in three-dimensional (3D) environments and behave as they would in the real world (verisimilitude). The most widely used types of VR technology are immersive VR, desktop VR, projective VR, and C-automatic virtual environment (CAVE). All of these types of VR aim to create life-like environments for training or assessment purposes.

“Immersion and interaction” are considered the two key criteria for classifying VR systems (Fuchs et al., 2011; Lenormand and Piolino, 2022) (Table 1). Two main types of VR immersion are reported (Kaplan-Rakowski and Gruber, 2019): low immersion virtual reality (LiVR) and high immersion virtual reality (HiVR). LiVR is defined as “a computer-generated three-dimensional virtual space experienced through standard audio–visual equipment, such as a desktop computer with a two-dimensional monitor” (*ibid* p. 553). An example of LiVR is the use of serious games, which are digital media applications designed primarily for educational purposes (Grossard et al., 2017). In contrast, HiVR is described as “a computer-generated 360° virtual space that can be perceived as being spatially realistic, due to the high immersion afforded by a head-mounted device” (*ibid* p. 553). While both LiVR and HiVR can be considered immersive, the degree of immersion varies significantly. In a highly immersive VR environment, the user should experience a strong sense of presence within the computer-generated scenario (Ip et al., 2018). Thus, the VR environment, apart from the multi-sensory stimulations, must provide users with possibilities for interaction. Kaplan-Rakowski and Gruber (2019) argue that the level of immersion is primarily determined by the technological interface: systems using a standard two-dimensional monitor, keyboard, or mouse are categorized as low-immersion, whereas those employing head-mounted displays or VR headsets are classified as high-immersion systems.

**Table 1:** Principal characteristics of virtual reality environments.

**Characteristic**	**Type**	**Definition**
Immersion	Low/high	Refers to technology-related aspect of virtual environments, such as audiovisual equipment which determine the extent to which VR systems can deliver immersive experiences (Rose et al., 2018). □**Low immersion:** Typically involves the use of a desktop computer with a two-dimensional monitor. □**High immersion:** Involves the use of head-mounted devices that provide a more encompassing and realistic sensory experience.
Interaction	Low/high	**Equipment allowing participant control of interaction within the virtual environment** • **Low interaction:** Navigation and actions are controlled via buttons or keyboard inputs. • **High interaction:** Navigation and actions are controlled through advanced tracking technology, such as three-dimensional tracking sensors that capture user movements.
Sense of presence	Spatial, self-, social-	Participant's subjective perception or feeling of truly being immersed within the virtual environment.
User perspective	1st PP, 3rd PP	Mental representations of events occurring within the virtual environment 1PP: participant see the event from his “own eyes” 3PP: participant see himself in the event from an observer's point-of-view
Embodiment	Self-presence + sense of self-location and sense of agency	Representation and subjective experience of one's body within a virtual environment

**Interaction** refers to a participant's ability to actively engage with and influence the virtual Environment (VE), thereby assuming a more or less active role within it (Lenormand and Piolino, 2022). Specifically, it denotes the participants' capacity to control their interaction with the VE. Similar to immersion, VE can be divided into two categories: High Interaction (e.g., using three tracking sensors) and Low Interaction (e.g., participants use buttons).

Another central concept in VR is the participant's subjective feeling of truly being, acting, and behaving within the virtual environment, commonly referred to as **presence** (Sanchez-Vives and Slater, 2005). The sense of presence is strongly influenced by both the level of immersion and the degree of interaction afforded by the system. A three-dimensional categorization of presence is proposed by Lee (2004): (a) spatial presence, (b) self-presence, and (c) social presence.

Finally, VR systems enable experimenters to manipulate participant **embodiment** and **user perspective** (*first-person vs. third-person perspective*). Embodiment refers to the representation of the body within the virtual environment and is closely related to the concept of *self-presence* (Gorisse et al., 2017) as well as to the sense of self-location and **agency** (Kilteni et al., 2012). Finally, we frequently experience and mentally represent events from different perspectives. For instance, autobiographical memories can be recalled either from a first-person perspective, where events are seen through one's own eyes, or from a third-person perspective, where one views oneself from an observer's standpoint (Iriye and St Jacques, 2021). Manipulating the participant's point of view in VR relies not only on technical factors such as camera positioning but also appears to be influenced by the level of immersion, with embodiment experiences differing markedly between low-immersion (LiVR) and high-immersion (HiVR) environments.

### 2.3 VR training/rehabilitation in children with ADHD or ASD

The application of artificial intelligence (AI) tools and techniques in populations with autism spectrum disorder (ASD) and attention-deficit/hyperactivity disorder (ADHD) has been the subject of several review studies (Cibrian et al., 2022; Lakes et al., 2022; Mazon et al., 2019). These reviews underscore the potential of AI-based approaches for both diagnosis and intervention, spanning a range of domains from mental health mobile applications and machine learning algorithm-based screening tools to social robots or virtual coaches targeting emotion regulation or non-social communication. However, the wide variety of IA-based methodologies combined with concerns about study quality (e.g., randomization procedures and ecological validity) presents significant challenges for drawing consistent and generalizable conclusions.

The framework of Virtual Reality-cognitive rehabilitation was first proposed by Rizzo and Buckwalter (1997) and tested with children with ADHD. More recently, Wang and Reid (2013) introduced an interactive, cognitive intervention for autism integrating traditional cognitive rehabilitation (specific and repetitive training exercises targeting impaired cognitive functions) with Virtual Reality technology.

Unlike traditional rehabilitation procedures, VR-based interventions enhance participant *engagement* and help sustain *attention* throughout the session owing to the flexibility of virtual environments, which can be dynamically modified and personalized to match individual characteristics (Table 2) or manipulate the degree of complexity (Wang and Reid, 2013). For example, in a VR classroom (Rizzo et al., 2000), children are immersed in a first-person perspective *using* a head-mounted display within a virtual environment that closely replicates a familiar classroom setting. Although the environment is designed to appear naturalistic, the number and characteristics of virtual characters (one teacher and several students) as well as the type and frequency of distractors are pre-determined by the experimenters. This controlled yet realistic setting was specifically developed to evaluate and train attentional skills in children.

**Table 2:** Advantages of using virtual reality training environments with neurodevelopmental disorder population.

**Advantages**	**Definition**
Immersiveness and realism	Use of realistic virtual environment enhancing participant's engagement and ecological validity
Targeted training program	Adaptation of stimuli and experimental conditions within the virtual environment to align with the individual characteristics, needs or cognitive profile of the participant
Experimental control	Manipulation of experimental variables within a controlled environment

Virtual environments also offer participants opportunities for realistic and dynamic engagement in the practice of social scenarios, making them particularly effective for individuals with neurodevelopmental disorders such as ADHD (Bashiri et al., 2017; Parsons et al., 2007) or ASD (Didehbani et al., 2016; Kandalaft et al., 2013). An emblematic example is the virtual reality—social cognition training (VR-SCT) program, which targets socioemotional and sociocognitive abilities in adults with ASD (Kandalaft et al., 2013). In this intervention, participants engage in “real-time” conversations with a live coach who asks questions related to the social scenario (fostering situational awareness) and provides immediate feedback on the participant's behavior.

Recent research indicates a significant association between Theory of Mind (ToM) and autobiographical memory (AM) (Duval et al., 2009; Frith and Frith, 2007), as both cognitive domains share overlapping neural substrates (Spreng and Grady, 2010) and contribute to social understanding (Corcoran, 2000). Individuals often rely on AM to interpret and navigate social scenarios by recalling relevant personal experiences. Virtual reality (VR) role-play scenarios have the potential to activate AM, thereby enhancing ToM performance (Schöne et al., 2019) through increased realism, embodiment (e.g., first-person perspective), and a strong sense of presence. These immersive features not only facilitate cognitive processing, promoting a shift from reactive to reflective reprocessing (Zelazo, 2015), but also improve participant engagement and motivation, which are critical for the effectiveness of interventions targeting neurodevelopmental disorders.

Lastly, adopting an ontogenetic perspective, virtual reality (VR) interventions offer the possibility to scaffold training by targeting basic Theory of Mind (ToM) or executive function (EF) skills before progressing to more advanced capacities. As proposed by Frith and Frith (2006), the distinction between top–down and bottom–up processing—originally applied to non-social cognitive domains—may be highly applicable to social cognition. While social stimuli can trigger automatic responses via bottom–up mechanisms, these responses can also be modulated through deliberate, top–down strategies, particularly when guided by explicit instruction. In this context, VR training programs may begin with foundational sociocognitive skills such as eye gaze, imitation, and emotion recognition (Fletcher-Watson et al., 2014), and gradually advance to more complex mentalizing abilities, including understanding intentions, distinguishing between real and apparent emotions, and attributing false beliefs.

### 2.4 Purpose of this review

Digital tools appear to be helpful in training both attentional or executive functions and socioemotional skills (Cobb et al., 2010). A number of technology-based interventions or assessment tools have been specifically designed for the pediatric population, including children with Attention Deficit Hyperactivity Disorder (Bashiri et al., 2017) and Autism Spectrum Disorders (Mazon et al., 2019; Wass and Porayska-Pomsta, 2014; Wang and Reid, 2011). These interventions often target specific domains of social interaction (Grossard et al., 2017) and have demonstrated positive and beneficial outcomes.

Although several reviews have already been conducted on specific social interactions using VR technology in individuals with ASD, the current review introduced two key objectives that extend beyond existing literature. Firstly, it aims to examine VR-based environments designed for the assessment and training of EF and ToM in populations with neurodevelopmental disorders. Therefore, we will consider the efficacy of these technology-based interventions in terms of reliability, consistency, durability, and generalization. While recent studies have highlighted the promise of VR, they often fail to specify the level of task complexity (basic, moderate, or complex skill) or to identify which features of the VR systems, such as immediate performance feedback, ecological validity, sense of presence, or degree of immersion, contribute most to their effectiveness. Second, the review aims to analyze how the sense of presence and immersive experience, the user's perspective, the interactive properties, ecological validity, and the participant's engagement are implemented within current paradigms. The methodological quality of the reviewed studies will then be assessed based on criteria including sample size, use of control groups, randomization, follow-up measures, and the ecological validity of outcome assessments in both training and evaluation contexts.

## 3 Methods

### 3.1 Inclusion procedure

**Figure 1:**
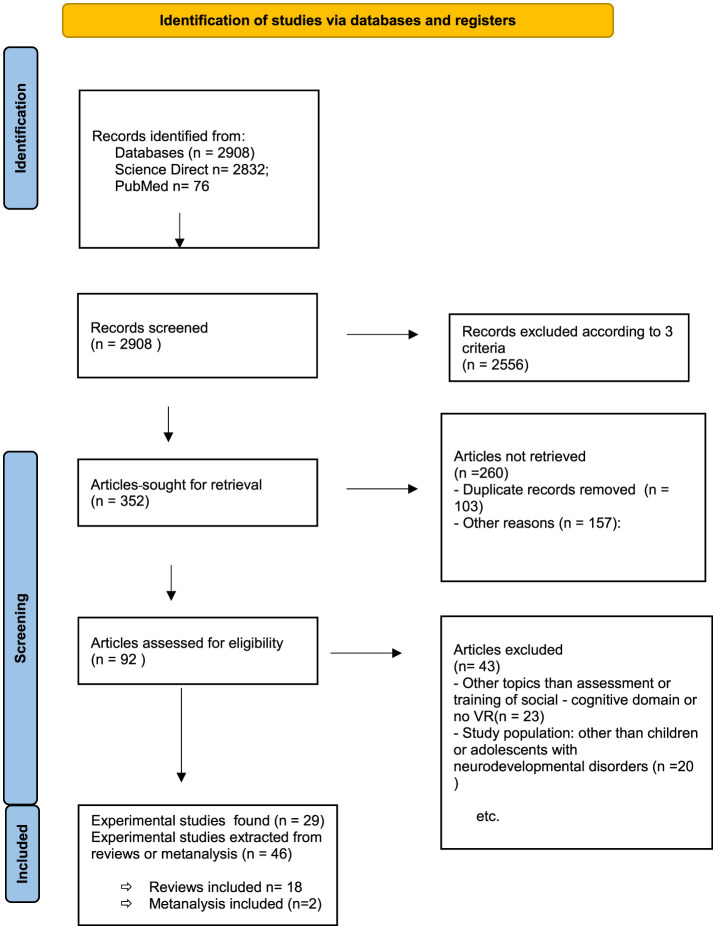
Flow diagram describing the paper selection process.

We reviewed the available literature on PubMed and Science Direct databases published between 1996 and 2022. The databases were screened with the key words “theory of mind,” “social training” OR “Executive Functions” AND “neurodevelopmental disorder” OR “autism” OR “ASD” OR “ADHD” AND “virtual reality.” The titles (records identified from Databases *n* = 2,908), abstracts (articles sought for retrieval *n* = 352; articles not retrieved *n* = 260), and full texts of relevant articles (articles assessed for eligibility *n* = 92; articles excluded *n* = 43) were reviewed for inclusion. The 75 studies included (experimental studies found *n* = 29; experimental studies extracted from reviews or metanalysis *n* = 46) in the analysis met the following criteria: (i) they reported on virtual environments developed for the assessment or training of social or executive skills; (ii) they reported on individuals with ADHD or ASD; (iii) they targeted a pediatric population (children or adolescents). We excluded all virtual reality protocols that were cited in reviews and where the original article was not accessible (see Figure 1).

### 3.2 Data extraction

For each article, general information about the study's purpose, population, and main results was extracted. We next examined each study's methodological dimensions, recording whether the virtual environments were used for assessment or training as well as the target domain (ToM or EFs). In addition, information about the population (ADHD or ASD, sample size, and age), the study's experimental research design (presence or not of a control group), as well as details of each training program (duration, number of sessions, presence of feedback, type of feedback, and modulation of degree of complexity) was extracted.

In addition to collecting demographic and methodological information, we examined and documented the characteristics of each VR environment. Specifically, we noted the following elements:

- **Degree of immersivity** (from low to high), depending on the equipment used for the VR experience.
- **Interactive properties**, that is, the participant's capacity to control interaction with the VE (High vs. Low). Under the term interaction, we explored **the participant's level of interaction with the VE** (e.g., interacting with peers/adults/a coach in the VR environment or before/after each VR session). Participants' degree of control based on the equipment used (e.g., joystick) was not taken into consideration.
- **Sense of presence and immersive experience:** this is a combination of two factors: immersivity and interaction. Description of cues presented in the VR environment, such as visual cues (e.g., panoramic 3D displays), auditory cues (e.g., surround sound acoustics), tactile cues (e.g., haptics and force feedback), olfactory and gustation cues (e.g., smell replication and taste replication).
- **User's point of view:** First-person or third-person perspective.
- **Ecological validity:** Use of real-world scenarios, settings, etc.
- **Participant's engagement and motivation:** If and how authors tried to measure participants' engagement and motivation during the VR training or assessment procedure.

## 4 Results

### 4.1 Overview of studies

A total of 75 studies (3 without data presentation) were included in the review. These studies described the use of innovative Virtual Reality environments for the assessment and/or training of cognitive or social skills in children or adolescents with neurodevelopmental disorders. Information about each study's objective, target population, and main findings are presented in Supplementary Table 1. Eighteen review articles and two meta-analyses (20 articles) are included in Supplementary Table 2.

### 4.2 Methodological dimensions of the studies

#### 4.2.1 Target skills/processes and clinical populations

The literature includes numerous studies in which VR has been employed both as an assessment tool and as a training program. A total of 16 studies using virtual environments for assessment purposes were identified, including 11 targeting EFs and five focusing on social skills. The VR environments designed to assess EFs primarily evaluated attentional skills in participants with ADHD. No protocols were identified that specifically targeted working memory, cognitive flexibility, or inhibition through VR environments. Within the domain of social cognition, all five studies focused on participants with ASD. Only Mundy et al. (2016) study explored both children with ASD and children with ADHD. Regarding the target areas, three studies using VR environments assessed specific social skills such as emotion recognition (Kim et al., 2015), joint attention (Mundy et al., 2016), or visual face exploration (Grynszpan et al., 2012). Two VR environments aimed to assess more general social abilities (Jung et al., 2006) or embodied social presence (Wang et al., 2016). Several inconsistencies were noted in the assessment of executive functions (EFs), as all identified protocols focused exclusively on attentional skills, without addressing a more comprehensive evaluation of participants' cognitive profiles. Additionally, there was a notable lack of studies assessing sociocognitive abilities within virtual reality (VR) environments prior to the implementation of training interventions.

A total of 59 **training studies** of cognitive or social skills were found, the majority focusing on improvement in social areas, as 46 out of the 59 targeted basic or more complex social skills. VR has also been used to train cognitive functions (Benzing and Schmidt, 2017; Bul et al., 2018, 2016; Chen et al., 2022; de Vries et al., 2015; Dovis et al., 2015; Skalski et al., 2021; Weerdmeester et al., 2016) and more precisely attentional processes (Cho et al., 2002, 2004; Lee et al., 2001; Parsons et al., 2004; Yan et al., 2008). Concerning social cognition, 18 studies targeted exclusively bottom–up processes such as emotion recognition (Bekele et al., 2014; Bölte et al., 2002, 2006; Deriso et al., 2012; Faja et al., 2007; Fernandes et al., 2011; Gordon et al., 2014; Grynszpan et al., 2008; Lacava et al., 2007; Liu et al., 2017; Rice et al., 2015; Serret et al., 2014; Tanaka et al., 2010; Williams et al., 2012), joint attention (Cheng and Huang, 2012; Mundy et al., 2016; Ravindran et al., 2019), and social attention (Amaral et al., 2017).

Considering that real-life social situations require the integration of cognitive, executive, and top–down social processes such as cognitive flexibility or perspective taking (Grossard et al., 2017), many training studies have focused on more complex social skills, including emotion regulation and social interaction (Ke et al., 2022; Yuan and Ip, 2018), social communication–collaboration (Abirached et al., 2011; Bauminger et al., 2007; Bauminger-Zviely et al., 2013; Fletcher-Watson et al., 2016), social collaboration-perspective taking (Parsons, 2015), ToM (Rajendran and Mitchell, 2000; Swettenham, 1996), interaction and communication (Ke and Im, 2013), emotional understanding and social skills (Beaumont and Sofronoff, 2008), social problem-solving abilities (Bernard-Opitz et al., 2001), social understanding (Mitchell et al., 2007), social cognition (Didehbani et al., 2016), or emotional (Frolli et al., 2022) and social adaptation skills (Ip et al., 2018). Finally, in some studies, the degree of complexity of the training was progressively modulated over the sessions, with the training or rehabilitation program first targeting basic processes and then gradually addressing more complex processes that are involved in social cognition (Hopkins et al., 2011; Moore et al., 2005; Silver and Oakes, 2001;Vahabzadeh et al., 2018).

Findings across the studies did not demonstrate a comprehensive assessment of functional ToM or EF encompassing the full spectrum from lower to higher-level processes as suggested by theoretical frameworks. Furthermore, the studies did not address the critical developmental period for the evolution of EF or ToM. Finally, despite ongoing debates regarding the relationship between ToM and EF (i.e., the emergence vs. the expression account), this question remained unexamined in the reviewed studies.

#### 4.2.2 Type of experimental research design and training characteristics

Among studies including a training program, various research designs were reported, ranging from single-group clinical trials to randomized controlled trials:

- A single group of children and adolescents with a neurodevelopmental disorder. In this case, majority of studies included a small number of individuals (Abirached et al., 2011; Bauminger et al., 2007; Cheng and Huang, 2012; Fernandes et al., 2011; Herrera et al., 2008; Ke and Im, 2013; Ke et al., 2022; Lacava et al., 2007; Lahiri et al., 2012; Liu et al., 2017; Mitchell et al., 2007; Parsons et al., 2004; Ravindran et al., 2019; Yan et al., 2008; Vahabzadeh et al., 2018; Wang et al., 2016). There were, however, four studies including a sample size of ≥ 20 participants (Bauminger-Zviely et al., 2013; Benzing and Schmidt, 2017; Didehbani et al., 2016; Moore et al., 2005).
- Two clinical subgroups or a clinical population group compared to a control group:
  ○ A group of participants with neurodevelopmental disorder (ADHD or ASD), whose performance was assessed before training (pre-test condition). Half of the participants were assigned to receive the intervention (training group) and half were included in a control group (non-training group). The performance of the two groups was reassessed after completion of the training sessions, at post-test (Beaumont and Sofronoff, 2008; Bölte et al., 2002; Fletcher-Watson et al., 2016; Frolli et al., 2022; Ip et al., 2018; Lee et al., 2001; Lorenzo et al., 2016; Rice et al., 2015; Silver and Oakes, 2001; Yuan and Ip, 2018; Weerdmeester et al., 2016; Williams et al., 2012). Sample sizes range from 10 (Bölte et al., 2002) to approximately 100 participants (Ip et al., 2018; Yuan and Ip, 2018) or more than 100 participants (Bul et al., 2016, 2018).
  ○ A group of participants with neurodevelopmental disorder and a group of healthy controls (Amaral et al., 2017; Bernard-Opitz et al., 2001; Grynszpan et al., 2008; Jung et al., 2006; Parsons, 2015). Majority of studies included a sample size of less than 20 participants, but a few had more participants (Bekele et al., 2014; Gordon et al., 2014).
- A randomized controlled trial including more than two groups of participants:
  ○ Two clinical groups receiving the training intervention in a VR environment or in a classical device and one control group (Cho et al., 2002).
  ○ One group included participants with a neurodevelopmental disorder, one included participants with another disorder (Down's Syndrome), and one included healthy participants (Swettenham, 1996).
  ○ Three clinical groups receiving different trainings (de Vries et al., 2015; Dovis et al., 2015; Skalski et al., 2021).

Concerning the assessment of EFs and social cognition through VR environments, research designs involved included (1) a single group of participants with neurodevelopmental disorder (Pollak et al., 2010; Wang et al., 2016); (2) a comparison between a group of participants with neurodevelopmental disorder and a group of healthy individuals (Adams et al., 2009; Bioulac et al., 2012; Gutiérrez-Maldonado et al., 2009; Grynszpan et al., 2012; Kim et al., 2015; Negut et al., 2017; Parsons et al., 2007; Rizzo et al., 2000; Yeh et al., 2012); and (3) two clinical groups and a control group of healthy participants (Mundy et al., 2016) or one clinical group and one control group of healthy participants as well as two assessment conditions (Rodríguez et al., 2018).

Considerable variability was observed in the duration of training programs, with the number of sessions differing widely across studies. In some studies, the training procedure was completed after only one session (Liu et al., 2017; Vahabzadeh et al., 2018) while in others the number of sessions reached 24 (Benzing and Schmidt, 2017), 25 (de Vries et al., 2015; Dovis et al., 2015), 28 (Herrera et al., 2008; Ip et al., 2018) or more than 30 (Bul et al., 2016, 2018; Frolli et al., 2022; Ke et al., 2022). In majority of studies, however, a more intermediate rate of training was preferred, with training completed after 6 (Rajendran and Mitchell, 2000; Weerdmeester et al., 2016; Yuan and Ip, 2018), 8 (Beaumont and Sofronoff, 2008; Chen et al., 2022; Cho et al., 2002, 2004; Faja et al., 2007; Swettenham, 1996), 10 (Bauminger et al., 2007; Bernard-Opitz et al., 2001; Didehbani et al., 2016; Jung et al., 2006; Lorenzo et al., 2016; Silver and Oakes, 2001; Skalski et al., 2021) or 14 sessions (Ravindran et al., 2019). The majority of training programs provided participants with various feedback sessions during the training procedure. Participants could, for instance, receive feedback from the trainer during the sessions (guidance and support) as well as before and after the training procedure (Yuan and Ip, 2018). In other studies, real-time visual (Bölte et al., 2002; Moore et al., 2005) or auditory feedback (Hopkins et al., 2011; Silver and Oakes, 2001; Weerdmeester et al., 2016), or both types of feedback (real-time visual and auditory feedback) were preferred (Bernard-Opitz et al., 2001; Liu et al., 2017). Feedback was used not only as a reinforcement in the case of a correct answer (Hopkins et al., 2011), but also as a hint in the event of an incorrect answer (Moore et al., 2005). In the study by Didehbani et al. (2016), each training session, lasting about 10 min, was followed by a 5-min feedback/discussion from the “coach” clinician. In a large number of articles, the presence or absence of corrective feedback, as well as their characteristics, were not explicitly described.

#### 4.2.3 Methodological analysis of study quality

The methodological quality of the included studies was evaluated based on the following criteria: sample size (>30 participants for studies with two groups, >20 for single- group studies), inclusion of a control group (e.g., clinical population vs. typically developing children), randomization (applicable only to training studies, comparing intervention and no-intervention groups), follow-up measures, and the ecological validity of outcomes measures. For the ecological validity of outcomes, we took into consideration the verisimilitude (level of resemblance between cognitive demands of a test and a real-life situation/environment) and the veridicality approach (level of correlation between existing tests and measures of everyday functioning). Each study received 1 point for each of 4 (assessment) or 5 (training) criteria.

Few studies achieved a total quality score of 3 or higher out of 4 or 5, indicating the predominance of feasibility or pilot studies (with promising results) and the relative absence of studies employing robust experimental designs. A detailed overview of the methodological characteristics of studies that achieved a high score, including targeted skills/processes, clinical populations, experimental designs, and training features, is provided in Table 3 (assessment) and Table 4 (training). Information about all 74 studies is presented in the Supplementary Tables 3, 4. Analysis of these tables reveals several noteworthy findings as presented in the following.

**Table 3:** Presentation of TOM or EF assessment studies' quality (*N* = 5/16).

**Authors**	**Research design: Clinical (ASD and ADHD) and Typical development (TD) population/sampling/age**	**Procedure: Assessment (duration, VR or non-VR task, and neuropsychological tests) Training (duration, VR or non-VR training, number of sessions, and type of feedback)**	**Evaluation study quality**
Adams et al. (2009)	Population: *N* = 35 • Clinical: 19 ADHD (boys) • TD: 16 age-matched TD• Age: 8–14 years.	**1. Assessment of attention (VR or non-VR task):** • Standard continuous performance task (The Vigil Psychological Corporation • Virtual reality classroom version of a continuous performance task **VR-CPT was administered first**. **2. Other evaluations:** • The Simulator Sickness Questionnaire (SSQ; Kennedy et al., 1993) • Behavior Assessment System for Children (BASC, Reynolds and Kamphaus, 1998).	Sample size: 1 Use of control groups: 1 Follow-up measures: 0 Ecologically valid outcomes: 1 **Total: 3/4**
Bioulac et al. (2012)	Population: *N* = 36 • Clinical: 20 ADHD (boys) • TD: 16 Age: 7–10 years.	**1. Assessment related to the study's inclusion criteria:** • Conners' parents rating scale (CPRS) Child Behavior Check List **2. Assessment related to the study's principal goal (assessment of attention):** • Virtual Classroom (VC) • Continuous performance test (CPT II). **Other measures**: • State Trait Inventory Anxiety (STAI) • A 22-item cybersickness scale Virtual Reality Classroom.	Sample size: 1 Use of control groups: 1 Follow-up measures: 0 Ecologically valid outcomes: 1 **Total: 3/4**
Kim et al. (2015)	Population: *N* = 42 • Clinical: ASD *n* = 19 (13 boys and 6 girls)• Age: 11 years 1 month, standard deviation (SD) = 2.5 • Group control TD *n* = 23 (16 boys and 7 girls, Age: 11 years 5 months, SD = 2.3 Age range for both groups: 8–16 years.	**1. Assessment related to the study's inclusion criteria:** • High Functioning Autism Spectrum Screening Questionnaire (ASSQ; Ehlers et al., 1999; Posserud et al., 2006) • The Social Communication Questionnaire (SCQ, Berument et al., 1999; Corsello et al., 2007) • Social Responsiveness Scale (SRS, Constantino, 2004). The assessment is related to the study's purpose. • **Virtual reality assessment**: ° Virtual reality emotion sensitivity test (V-REST; Kim et al., 2010). **2. Other measures:** • Child version of the Reading the Mind in the Eyes (RME) task (Baron-Cohen et al., 2001). • Wechsler Abbreviated Scale of Intelligence (WASI) • Manifest Anxiety Scale for Children (MASC, March, 1997). • Behavior Assessment System for Children – 2 (BASC-II; Reynolds and Kamphaus, 2004).	Sample size: 1 Use of control groups: 1 Follow-up measures: 0 Ecologically valid outcomes: 1 **Total: 3/4**
Negut et al. (2017)	Population: *N* = 75 (45 boys and 30 girls) Age: 7–13 years • Clinical: ADHD = 33 Age: 10.24 years • TD: *N* = 42 Age: 8.9 years Two experimental assessment conditions: • VC • Traditional CPT.	**Assessment**: 2 conditions • Traditional assessment: continuous performance test (CPT) • ClinicaVR: Classroom – CPT (VC) • Variables measured in both conditions: • Total correct responses • Errors of commission • Errors of omission • Mean reaction time • Testing session lasted for approximately two hours. **1. Assessment related to the study's inclusion criteria:** • Romanian form of RavenStandard Progressive Matrices Plus (Dobrean et al., 2008; Domuţa et al., 2004) **2. Assessment related to the study's purpose:** • Digit Span and Letter Number Sequencing subtests, Coding and Symbol Search subtests Wechsler Intelligence Scale for Children – Fourth Edition (WISC-IV; Wechsler, 2003) • d2 Test of attention (Brickenkamp and Zillmer, 1998) **3. Other measures:** • Simulator Sickness Questionnaire (SSQ; Kennedy et al., 1993) • Cognitive Absorption Scale (CAS; Agarwal and Karahanna, 2000).	Sample size: 1 Use of control groups: 1 Follow-up measures: 0 Ecologically valid outcomes: 1 **Total: 3/4**
Rodríguez et al. (2018)	Population: *N* = 238 (241 boys and 97 girls) Age: 6–16 years (*M* = 10.84, SD = 3.01) • Clinical: ADHD = 237 • 31.95% inattentive presentation • 15.38% impulsive–hyperactive presentation • 22.78% combined presentation • TD = 101 Two experimental conditions: • Assessment with TOVA (traditional CPT): *n* = 172 (67.40% boys and 32.60% girls) Age: m = 10.55 years • Assessment with Aula Nesplora (VR-CPT): *n* = 166 (75.30% boys and 41% girls) Age: *M* = 11.10.	**Assessment:** • **2 conditions** • **VR CPT:** Aula Nesplora • **Traditional CPT: Test of Variables of Attention (TOVA)** **1. Assessment related to inclusion criteria:** • ADHD: Attention deficit hyperactivity disorder assessment scale (EDAH) (Farré and Narbona, 2003) • Intelligence quotient (IQ): Wechsler Intelligence Scale for Children-IV (WISC-IV) • Anxiety, depression, etc. for control group: (DISC-IV; Shaffer et al., 2000).	Sample size: 1 Use of control groups: 1 Follow-up measures: 0 Ecologically valid outcomes: 1 **Total: 3/4**

**Table 4:** Presentation of EF or TOM training studies' quality (*N* = 12/56).

**Authors**	**Research design: Clinical (ASD and ADHD) and Typical Development (TD) population/sampling/age**	**Procedure: Assessment (duration, VR or non-VR task, and neuropsychological tests) Training (duration, VR or non-VR training, number of sessions, and type of feedback)**	**Evaluation study quality**
Beaumont and Sofronoff (2008)	Population: *N* = 49 • Clinical: ASD • Intervention group ASD (*n* = 26) • Group control ASD (*n* = 23) Age: 7.5–11 years	VR Training: • Four components: group social skills training, parent training, teacher handouts, and a computer game (Junior detective computer game targeting emotion recognition, emotion regulation, and social interaction) • Sessions: 7 • Follow-up: 6 weeks and 5 months. 1. Assessment related to the study's inclusion criteria: • Childhood Asperger Syndrome Test (CAST; Scott et al., 2002) • Social Skills Questionnaire- teacher and parent Version (SSQ-P) • IQ = Short-form WISC-III 2. Assessment related to training: • Emotion Regulation and Social Skills Questionnaire (ERSSQ) • Assessment of Perception of Emotion from Facial Expression. • Assessment of Perception of Emotion from Posture Cues. • James and the Maths Test (Attwood, 2004a) Dylan is Being Teased (Attwood, 2004b).	Sample size: 1 Use of control groups: 0 Randomization: 1 Follow-up measures: 1 Ecologically valid outcomes: 1 Total: 4/5
Bul et al. (2016)	Population: *N* = 170 children Age: 8–12 years • Clinical: ADHD • Group 1: 88 ADHD participants received game intervention + usual treatment for the first 10 weeks. After 10 weeks, they received only the usual treatment for the next 10 weeks. Analyses for 68 participants. • Group 2: 82 ADHD participants received usual treatment for the first 10 weeks. After 10 weeks, they also received a serious game intervention for 10 weeks. Analyses for 71 participants.	Training: • Duration: 20-week. Participants received serious game intervention for only 10 weeks. Participants instructed to play the serious game for a maximum of 65 minutes (duration of each session), 3 times per week (total of 30 sessions). • VR training: Serious Game (Plan-It Commander), mission-guided game divided into 10 different missions and side missions. • Gratification: badges or medals in their profile, rewards (papercraft models, desktop wallpapers, and music). **1. Assessment related to the study's inclusion criteria:** • Kiddie Schedule for Affective Disorders and Schizophrenia-Lifetime version [K-SADS] • Disruptive Behavior Disorder Rating Scale (DBDRS) • Wechsler Intelligence Scale for Children III [WISC-III] **2. Assessment related to training** • Time management questionnaire • Plan/Organize the Behavior Rating Inventory of Executive Function (BRIEF – parent version and teacher version) • Cooperation of the Social Skills Rating System (SSRS – parent version) • Secondary outcomes • Subscale Working Memory of the BRIEF (parent and teacher version) • subscales Responsibility, Assertiveness, Self-Control, and Total of the SSRS (parent version and teacher version) • It's About Time Questionnaire (IATQ – parent version) • Self-efficacy questionnaire.	Sample size: 1 Use of control groups: 0 Randomization: 1 Follow-up measures: 0 Ecologically valid outcomes: 1 **Total: 3/5**
Bul et al. (2018)	Population: *N* = 143 (initially 170) Clinical: ADHD Age: Mean 9.90 years (SD = 1.26) • Intervention group ADHD (*n* = 88) • 10-week intervention: *n* = 73 • 20-week intervention: *n* = 68• Age: Mean • Control group ADHD (*n* = 82) a) 10-week intervention: *n* = 79 b) 20-week intervention: *n* =71.	Training: • Period of 20 weeks training (a) 10 weeks serious game intervention + usual treatment, (b) 10 weeks usual training). • 1 h session three times a week. Total sessions: 30 sessions Serious game: computer game “Plan-It Commander” **1. Assessment related to study's inclusion criteria:** • Wechsler Intelligence Scale for Children III -WISC-III (**Intelligence quotient)** • Kiddie-Schedule for Affective Disorders and Schizophrenia-Life- time version-K-SADS (**ADHD diagnosis)** • Disruptive Behavior Disorders Rating Scale- DBDRS (s**everity of ADHD symptoms)**. **2. Assessment related to training** • Measures were administered at baseline (T0), at 10 weeks (T1), and at 10-week follow-up (T2). • Behavior Rating Inventory of Executive Function -BRIEF (executive functions, planning/organizing skills) • Social Skills Rating System (SSRS) – parent version (cooperation skills) • Management questionnaire.	Sample size: 1 Use of control groups:0 Randomization: 1 Follow-up measures: 1 Ecologically valid outcomes:1 **Total: 4/5**
Cho et al. (2002)	Population: *N* = 26 • Clinical: ADHD = 26 (not officially diagnosed ADHD. Participants described as having learning difficulties, being inattentive, impulsive, hyperactive, and distracted) 3 groups: • VR Training group (*n* = 8) Age: 13 years • Non-VR Training group (*n* = 9) Age: 15.11 years • Control group (*n* = 9) Age: 14.67 years	**Training (VR and non-VR):** 8 sessions, about 20 min over 2 weeks (for the VR group and the non-VR group). • Two cognitive training courses: Virtual Reality Comparison Training Task and Virtual Reality Sustained Attention Training Task. • Same tasks for both groups, but in • VR training: use of HMD and head tracker and • Non-VR training: use of a computer monitor. 1. Assessment/Measures related to training: number of correct answers and response time. 2. Assessment based on neuropsychological evaluation: • Continuous performance task (CPT) before and after training sessions.	Sample size: 0 Use of control groups: 1 Randomization: 1 Follow-up measures: 0 Ecologically valid outcomes: 1 **Total: 3/5**
Cho et al. (2004)	Population: *N* =28 (boys). • Clinical: ADHD = 28 (participants not officially diagnosed, described as inattentive, impulsive, hyperactive, distracted, and having difficulties in learning)• Three groups: • Control group (*n* = 9) • VR group (*n* = 10) • Non-VR group (*n* =9) Age: 14–18 years	**Training:** • Sessions of neurofeedback training over 2 weeks. • Each session: approximately 20' Measures: **1. Assessment related to training** • **Continuous performance task (CPT): before and after training** ° Number of hits ° Reaction time ° Perceptual sensitivity ° Omission and commission errors ° Response bias **2. Other evaluations/measures** • EEG measurement.	Sample size: 0 Use of control groups: 1 Randomization: 1 Follow-up measures: 0 Ecologically valid outcomes: 1 **Total:3/5**
de Vries et al. (2015)	Population: Initially *N* = 166 applications, 132 screened, final *N* = 121 included Age: 8–12years • Clinical: ASD in three conditions ° Working Memory training: *n* = 40. Analyses for 31 participants ° Cognitive flexibility training: *n* = 37. Analyses for 27 participants ° Non-adaptive control training “Mocking training”: *n* = 38. Analyses for 32 participants	•Study's schedule: Screening, pre-training, post-training (after 6 weeks), and follow-up (after 6 more weeks). • Training: ° Duration: Total of 25 sessions; 6 training weeks. • VR training: “Brain game Brian” **1. Assessment related to the study's inclusion criteria:** • Social Responsiveness Scale parent report (SRS: Constantino et al., 2003; Roeyers et al., 2011) • Autism Diagnostic Interview Schedule-Revised (ADI-R: De Jonge and de Bildt, 2007; Lord et al., 1994) • Two subtests of the Dutch version of the Wechsler Intelligence Scale for Children (WISC-III: Kort et al., 2002; Sattler, 2001). **2. Assessment related to training:** • WM tasks resembling the training task: Corsi block tapping task (Corsi-BTT: Corsi, 1972) • Cognitive flexibility task resembling the training tasks: Gender-emotion switch task (Chapter 2: de Vries and Geurts, 2012) • WM task different from the training tasks: the n-back task (Casey et al., 1995; Smith and Jonides, 1999). • Cognitive flexibility task different from the training tasks: number-gnome switch task, an adaptation of the number-switch task (Cepeda et al., 2000) • Inhibition: adaptation of the classical stop task (Logan, 1994) • Sustained attention: Sustained attention response task (SART: Robertson et al., 1997)• Far-transfer to daily life (EF, Social behavior, ADHD characteristics) • The Behavior Rating Inventory of Executive Function (BRIEF: Gioia et al., 2000; Dutch Version: Smidts and Huizinga, 2010; 75 items, 3-point Likert scale) • The Children's Social Behavior Questionnaire (CSBQ, Dutch version: Hartman et al., 2007; 49 items, 3-point Likert-scale) • The Dutch parent version of the Disruptive Behavior Disorders Rating Scale (DBDRS: Oosterlaan et al., 2000; Pelham et al., 1992; 42 items, 4-point Likert-scale).	Sample size: 1 Use of control groups: 0 Randomization: 1 Follow-up measures: 1 Ecologically valid outcomes:1 **Total: 4/5**
Dovis et al. (2015)	Population: ADHD *N* = (89) Age: 8–12 years • Full-active condition (*n* = 31) Age: 10.6 (SD = 1.4) • Partially Active (*n* = 28) Age: 10.3 (SD = 1.3) • Placebo (*n* = 30) Age: 10.5 (SD = 1.3).	**VR training:** • Braingame “Brian” (BGB): computerized, home-based EF training. • Number of sessions: 25 • Duration of each session: 35–50 **1. Assessment related to study's inclusion criteria**: • Disruptive Behavior Disorder Rating Scale (DBDRS) • Diagnostic Interview Schedule for Children, parent version (PDISC-IV) • Dutch Wechsler Intelligence Scale for Children (WISC-III|) **2. Assessment related to training** • Stop task: stop signal reaction time (SSRT). • Stroop: The Stroop Color and Word Test • Corsi Block Tapping Task (CBTT) • Digit span: the Digit-span subtest from the WISC-III test battery. • Trail Making Test (TMT): of the Delis-Kaplan Executive Function System (D-KEFS) • Raven colored progressive matrices. • Behavior Rating Inventory of Executive Function questionnaire (BRIEF). • Sensitivity to Punishment and Sensitivity to Reward Questionnaire for children (SPSRQ-C). • Home Situations Questionnaire (HSQ).	Sample size: 1 Use of control groups: 0 Randomization: 1 Follow-up measures:0 Ecologically valid outcomes: 1 **Total:3/5**
Faja et al. (2007)	Population: *N* = 10 • Clinical: ASD = 10 • Training group (*n* = 5) • control group (*n* = 5) Age: 12–32 years	**Training:** • 8 training sessions during a 3-week period. • Session: 30 min to 1 h. • *Explicit rule-based instruction* emphasizing configural processing of faces • Post-test within a month **1. Assessment related to the study's inclusion criteria**: • Autism Diagnostic Interview–Revised (ADI-R) • Autism Diagnostic Observation Schedule (ADOS) • Abbreviated version of the Wechsler Intelligence Scale for Children–Third Edition (WISC–III) or the Wechsler Adult Intelligence Scale–Third Edition (WAIS–III). **2. Assessment related to training** • Standardized measures: ° Long form of the Benton Test of Facial Recognition (1983) ° Faces subtests of Wechsler Memory Scale–Third Edition (WMS–III) or Children's Memory Scale. • Self-report of face-processing ability • Experimental measures -materials presented on laptop. Face stimuli (black-and-white photos). The faces used in each experimental condition differed from those used in the training.	Sample size: 0 Use of control groups: 0 Randomization: 1 Follow-up measures: 1 Ecologically valid outcomes:1 **Total: 3/5**
Hopkins et al. (2011)	Population: *N* = 51 (final 49 as two participants were excluded). • Clinical: ASD = 49 (5 girls and 44 boys)• Four conditions: • Low-Functioning Autism (LFA) training (*N* = 11) • Low-Functioning Autism (LFA) control (*N* = 14) • High-Functioning Autism (HFA) training (*N* = 13) • High-Functioning Autism (HFA) control (*N* = 11) Age: 6–15 years	•**Training (VR and non-VR):** • Control (art Software): 12 (2 sessions per week × 6 weeks). Each session lasts approximately 10–25 min. • Experimental (FaceSay software): 12 (2 sessions per week × 6 weeks). Each session lasts approximately 10–25 min. • FaceSay software contains three different Games. • Feedback by coach avatar (e.g., “Good Job”) • Post-test measures: completed within 2 weeks. **1. Assessment related to the study's inclusion criteria:** • Evaluation: Childhood Autism Rating Scale (CARS) • Kaufman Brief Intelligence Test, Second Edition (KBIT). **2. Assessment related to training:** • Emotion Recognition: both photographs (Unmasking the Face) and schematic drawings, Benton Facial Recognition Test (Short Form), Social Skills Observation, Social Skills Rating System (SSRS) • Social Skills Observation.	Sample size: 1 Use of control groups: 0 Randomization: 1 Follow-up measures: 0 Ecologically valid outcomes:1 **Total: 3/5**
Ip et al. (2018)	Population: *N* = 114^*^ • Clinical: ASD *N* = 94 (86 boys and 8 girls) Age: 6–12 years • Group 1: (Training): 42 boys and 5 girls • Group 2 (Control): 44 boy participants and 3 girl participants• Pilot group of 20 children (to test out the design of the scenarios)	**Training:** • 28-session program that lasted for 14 weeks. • Training in three stages: briefing, VR-enabled training, and debriefing. • 3–4 children (similar age) participate in each session together. • **VE sessions** last 40 min: 10 min of direct exposure to the VR environment and 30 min of observation. **1. Assessment related to the study's inclusion criteria:** • Raven's Progressive Matrices (RPM) (Raven et al., 1998) • Childhood Autism Spectrum Test (CAST) (Williams et al., 2005) **2. Assessment related to training:** • Faces Test • Eyes Test (Psychoeducational Profile, Third Edition (PEP-3) (Schopler et al., 2004) • Adaptive Behavior Assessment System, Second Edition (ABAS-II)	Sample size: 1 Use of control groups:0 Randomization: 1 Follow-up measures:0 Ecologically valid outcomes: 1 **Total: 3/5**
Parsons et al. (2004)	Population: *N* = 36 • Clinical: ASD = 12 (10 boys and 2 girls) Age: 13–18 years • TD: *N* = 24 TD^*^^*^Each ASD participant was matched with two other pupils • one matched on verbal IQ and • the other matched on performance IQ	**VR training:** • VR program: Virtual *Café* (after completing four training trials) **1. Assessment related to the study's**_**inclusion criteria:** • Abbreviated Scale of Intelligence; Wechsler, 1999) • CAT; NFER **2. Assessment related to training:** • Behavioral Assessment of the Dysexecutive Syndrome (BADS; Wilson et al., 1986).	Sample size: 1 Use of control groups: 1 Randomization: 0 Follow-up measures: 0 Ecologically valid outcomes: 1 **Total: 3/5**
Rice et al. (2015)	Population: *N* = 31 (28 boys) Age: 5–11 years (M = 7.77) • Clinical: *n* = 31 ASD • Training/intervention group: *n* = 16 (boys) Age: M = 7.68 (SD = 1.45) • Group control: *n* = 15 (12 boys and 3 girls) Age: M = 7.87 (SD = 1.60).	VR training: • *FaceSay* computer program (emotion recognition, emotions, understanding of others; perspectives, social skills)*a)* “*Amazing Gazing” game targeting eye gaze and responding to joint attentionb)* “*Follow the Leader” game targeting facial expressions of emotions in avatars*. Group control training: *SuccessMaker^®^* • Duration: NA **1. Assessment related to inclusion:** • WISC-III or WISC-IV **2. Assessment related to training:** • Affect recognition (NEPSY-II, Korkman et al., 2007). • Theory of Mind (NEPSY-II, Korkman et al., 2007). • Social Responsiveness Scale, Second edition (SRS-2; Constantino and Gruber, 2002) • Observation of a) positive interactions (total number): when participant initiated and engaged in positive interactions with a peer (“direct eye contact, direct eye contact combined with a smile; a smile with no eye contact, an expression of affection delivered verbally or non-verbally, etc.”) and b) negative interactions (total number): when participant engaged in negative interactions with a peer (“physical or verbal aggressiveness, etc”).	Sample size: 1 Use of control groups: 0 Randomization: 1 Follow-up measures: 0 Ecologically valid outcomes: 1 **Total:3/5**

**Sample size:** A total of 11 out of 16 assessment studies included more than 30 participants. In contrast, this was not the case for the majority of training studies, limiting the generalizability of their findings.

**Control group:** A total of 13 out of 16 assessment studies incorporated a control group as part of the experimental procedure. In contrast, only 12 out of 59 training studies included a typically developing participant control group.

**Randomization:** Among the 59 training studies, 24 employed a randomization procedure for the clinical population. Notably, only three studies combined randomization with the inclusion of a control group.

**Follow up measures:** Only six studies proposed follow-up measures.

**Ecologically valid outcomes:** We also collected information on the tests/tasks administred to participants during the assessment session and before/or after the training procedure. Notably, in studies using VR-classroom environments, many authors chose to compare the effectiveness of VR with traditional measures such as the continuous performance test (CPT). In training studies, questionnaires like the Behavior Rating Inventory of Executive Function (BRIEF) were often administered. However, this was less common in the domain of social cognition training, where few studies validated their outcomes using standardized tools such as NEPSY-II (Didehbani et al., 2016) or questionnaires. In studies involving ASD populations, diagnostic tools such as the Autism Diagnostic Interview–Revised (ADI-R) and the Autism Diagnostic Observation Schedule (ADOS-2) were primarily used for participant recruitment rather than outcome validation. Finally, studies assessing attentional capacities seldom included broader evaluations of other cognitive domains such as inhibition, memory, or cognitive flexibility (de Vries et al., 2015; Benzing and Schmidt, 2017; Ke et al., 2022).

### 4.3 Virtual reality characteristics

#### 4.3.1 Virtual reality tools: a wide combination of technologies

Only a limited number of studies provided young participants with a high immersion VR experience, either for the assessment (Negut et al., 2017; Parsons et al., 2007; Rizzo et al., 2000; Rodríguez et al., 2018; Yeh et al., 2012) or the training of cognitive (Benzing and Schmidt, 2017; Skalski et al., 2021) or social skills (Cho et al., 2002, 2004; Lee et al., 2001). The majority of these studies targeted ADHD participants, with few studies focusing on the ASD population (Amaral et al., 2017; Ip et al., 2018; Lorenzo et al., 2016; Ravindran et al., 2019). Regarding the immersive experience, majority of studies mainly used visual and/or auditory cues. Three HiVR studies, besides visual and auditory, also proposed tactile cues (Cheng and Huang, 2012; Jung et al., 2006; Lacava et al., 2007). No studies using smell (olfactory) or taste replication (gustation) elements were found.

#### 4.3.2 VR interactive properties

In many studies, the VR environment was described broadly as a “scenario” or an “interactive environment” without clearly distinguishing the variability concerning its **interactive properties**. To illustrate this variability/diversity, the following sub-section provides a comparative analysis of two VR environments used respectively for assessment and training purposes. Supplementary Table 3 presents the interactive properties of all the studies as well as the opportunity for users to be engaged in social interactions within the VE.

#### 4.3.3 VR interactive properties during assessment

Rizzo et al. (2000) designed a VR-classroom to assess attentional skills in children with ADHD. The scenario simulated a realistic classroom with a blackboard, desks, a virtual teacher, and classmates. Participants received task instructions from the virtual teacher and could visually explore the environment using a mouse, although they had no navigational control. It remains unclear whether participants were embodied via avatars. While the authors described the system as an “interactive environment,” the interactivity was limited: participants did not engage in reciprocal interactions with virtual characters or manipulate objects within the environment.

In contrast to the VR-classroom, the study by Kim et al. (2015) employed a low-immersion environment (LiVR) to assess emotion sensitivity. Participants were presented with characters displaying one of six basic emotions through facial expression, body gesture, and verbal communication in a simulated real world (kitchen or living room). Although participants could not engage in contingent dialogue with the avatar, they were exposed to a naturalistic form of social interaction. Using a joystick, participants could adjust their proximity to the avatar and identify the emotions by selecting corresponding labels from a set of options on-screen. This setup enabled assessment of approach–avoidance motivation in relation to emotional stimuli, despite limited interactivity.

#### 4.3.4 VR interactive properties during training

In a single-user VE paradigm (LiVR) with ASD children (Mitchell et al., 2007), participants were trained to initiate interactions with virtual characters in a simulated *Café* setting. This scenario targeted several social learning objectives (initiating conversations), but interaction was limited to selecting avatars via mouse clicks.

Other studies targeting the same population (ASD) and communicative domain reveals considerable variability in the interactive properties of the VE. For example, Amaral et al. (2017) developed a LiVR paradigm using a P300-based Brain–Computer Interface (BCI) to train social attention. Their VE simulated a realistic child's bedroom containing furniture, objects, and an avatar. During training sessions, participants were instructed to observe the avatar and attend to the objects it turned its head toward. The participant interaction in this study was relatively passive, especially when compared to studies such as Didehbani et al. (2016).

#### 4.3.5 User's point of view: **first-person vs. third-person perspective**

The majority of HiVR protocols included in this review employed a first-person perspective (1PP). Gorisse et al. (2017) investigated first- and third-person perspectives in immersive virtual environments. Findings indicated that 1PP facilitated more precise interactions with virtual elements, whereas 3PP enhanced users' spatial awareness. Interestingly, despite their lower level of immersion, several LiVR programs also employed a first-person viewpoint. Exceptions include environments using fictional characters for participant embodiment, where third-person perspectives were adopted (e.g., Beaumont and Sofronoff, 2008; Weerdmeester et al., 2016).

### 4.4 Ecological validity

Not all studies included in this review provided detailed descriptions of the virtual environments (VEs) or the characteristics of the virtual characters presented to participants. However, a general trend is the use of VEs modeled on real-life settings, such as classrooms, homes, or public spaces. Following the seminal study by Rizzo et al. (2000), the virtual classroom paradigm has been extensively used for the assessment of attentional skills in ADHD populations (Bioulac et al., 2012; Pollak et al., 2010). Other real-life locations/places used as virtual environments are coffee shops (virtual café in Lorenzo et al., 2016; Parsons et al., 2004), a supermarket (Herrera et al., 2008), and a bedroom (Amaral et al., 2017). Fewer studies use multiple real-life locations (Bul et al., 2016, 2018; de Vries et al., 2015; Ke et al., 2022). For example, in the training protocol of Didehbani et al. (2016), various locations were proposed to users, such as a classroom, playground, and campground. We found two studies in which protocols were based on the principles of Augmented Reality (Escobedo et al., 2012; Vahabzadeh et al., 2018). In a few studies, authors pay attention to attributing real-life characteristics to avatars (Abirached et al., 2011). For instance, in Lorenzo et al. (2016), avatars' expressions change according to the participant's real-life expressions, due to a vision system.

#### 4.4.1 The sense of presence and immersive experience

Although participant engagement is reported as a key advantage of VR environments, relatively few studies have systematically evaluated participants' sense of presence or immersive experience. In the studies that did address this issue, subjective experience was evaluated using self-report measures such as the realistic subscale of the Presence Questionnaire, the adapted version of the UQO Cyberpsychology Laboratory (Nolin et al., 2016), or the subjective feedback questionnaire-SFQ (Pollak et al., 2010). In addition to questionnaires (Ravindran et al., 2019), interviews were used to capture participants' VR experience in more depth (Abirached et al., 2011; Bul et al., 2016; Ke and Im, 2013; Weerdmeester et al., 2016).

#### 4.4.2 Impact of VR features on study outcomes and transfer effects

An overview of virtual reality characteristics reported across 73 studies is presented in Supplementary Table 5. This subsection examines how the VR features—including the level of immersion, degree of interaction, user perspective, and embodiment—impact study outcomes and facilitate the transfer of executive and/or socioemotional skills beyond the virtual context.

Swettenham (1996) investigated the effectiveness of a computerized version of the Sally-Anne false belief task as a training tool in three groups: children with ASD, Down syndrome, and typically developing children. The computerized task was delivered in a **low-immersion virtual environment**, providing **visual cues** and a **first-person perspective**. Interaction was limited to **mouse-based navigation**. A follow-up assessment using classical false belief tasks was conducted 3 months after the intervention. The results showed that all groups passed near-transfer Tasks (a Dolls-based version of the Sally-Ann task) with no significant differences between groups. Moreover, training effects were maintained across all groups at follow-up. However, the ASD group exhibited persistent difficulties with far-transfer tasks (standard false belief tasks).

Bauminger et al. (2007) implemented a 10-session virtual training program designed to improve social communication skills in children with ASD. The intervention employed the Story Table interface, a **low-immersion environment** providing **visual and auditory cues** from a **first-person perspective**. The virtual setting included **non–realistic elements** (e.g., animated ladybugs) and participants interacted via **touch-screen activation of audio content**. The study reported positive outcomes, including improvements in social interaction—as evidenced by far-transfer effects on the **Marble Works task**—and a reduction in repetitive behaviors among the six participants. However, the absence of **randomization**, **a control group**, and **follow-up measures** limits the generalizability of the findings.

In their study, de Vries et al. (2015) evaluated the effectiveness of two executive function training programs (targeting working memory and cognitive flexibility) in children with ASD. The “Brain Game Brian” intervention was delivered through a low-immersion virtual environment, featuring visual and auditory cues. Real-life settings—such as a village or a beach—were simulated from a first-person perspective, with participants assuming the role of the character Brian and experiencing each scene through his point of view. Near-transfer effects were reported with improvements in working memory, cognitive flexibility, and attention, although no significant gains were found in inhibitory control. Additionally, the authors reported far-transfer effects to daily life, including improvements in BRIEF and social behavior scores and overall quality of life outcomes.

## 5 Discussion

Many systematic reviews and meta-analyses have already reported the efficacy of VR not only as a tool for cognitive and behavioral assessment but also as an intervention method for clinical populations. The present review extends this evidence by examining its application in pediatric populations with executive and/or sociocognitive impairments. Specifically, we aimed to provide a comprehensive overview of both the **clinical** and **VR features** of the included studies.

A total of 75 articles aiming to assess or train executive functions and/or social cognition in clinical populations were identified. Notably, the majority of training studies focused on individuals with ASD populations and targeted social skills. In contrast, studies using VR for assessment predominantly involved children with ADHD, with a focus on attentional skills.

While encouraging results regarding the efficacy of VR paradigms are reported, the heterogeneity of the studies, either in terms of experimental research design or the VR characteristics of programs, limits the comparison between protocols. Thus, the efficacy (reliability, consistency, durability, and generalization) of these interventions should be further explored.

VR immersion protocols provide participants with a multi-component, ecologically valid experience that simultaneously engages sensori-motor, cognitive, and/or social skills. The majority of studies favor a first-person perspective, as it typically enhances the user's sense of presence within the virtual environment. Improvements in targeted capacities may be attributed to the fact that exercises performed within a VR environment offer constantly increasing feedback, enabling the potential development of the participant's “awareness of the results” (meta-awareness), and thus metacognition. This development gradually promotes brain plasticity processes through complex mechanisms (De Luca et al., 2018). In line with the Iterative Reprocessing (IR) model (Zelazo, 2015), the use of avatars and a third-person perspective within VR environments may further enhance self-monitoring, attentional control, and facilitate the shift from reactive to reflective cognitive functioning.

As demonstrated in other clinical populations (e.g., Traumatic Brain Injury), the use of immersive VR technology is limited by issues of accessibility of technology and cost (Maggio et al., 2019). The development of a virtual environment (VE) can be time-demanding, resource-intensive, and dependent on digital literacy. Consequently, as highlighted in the present review, the majority of the authors prefer to develop LiVR environments. These limitations raise important questions regarding the potential of “serious games” in the assessment and training of sociocognitive skills. As noted by Maggio et al. (2019), serious games offer a low-cost alternative, enabling interactive virtual simulations in a controlled, safe environment while promoting the generalization of acquired skills. Serious game key features such as storylines, feedback, and increasing levels of difficulty are considered crucial to enhance learning outcomes. In contrast, traditional VR learning contents simulate highly specific social situations, which may restrict the transferability of trained skills to everyday contexts. Nevertheless, serious games have not yet achieved a high level of immersion. This raises important considerations for the design of serious game environments that incorporate not only visual and/or auditory cues, but also more complex immersive and interactive experiences (e.g., tactile cues and motion). This type of environment could be very promising for the ASD population. Additionally, even in the case of serious games, the design features of virtual environments are often insufficiently documented (use of the word “scenario” and the term “interactive VR program”), without providing detailed descriptions of the environment, nor arguments justifying the selection of specific features. Additionally, rarely reported measures about participants' motivation or consideration of negative elements related to VR, like cybersickness.

The data from this current review provide evidence of the efficacy of VR in the assessment and training of sociocognitive skills. These promising findings are yet to be confirmed by further studies that are more detailed in terms of experimental design (sample size, cross-sectional or long-term follow-up design, randomized-controlled trial, etc.). In the context of training programs targeting social skills in ASD populations, an additional limitation is the absence of an initial assessment. As Ip et al. (2018) indicated, many studies present outcomes following VR exposure without providing adequate information on pre- or post-intervention evaluation protocols. Moreover, long-term follow-up assessments are seldom reported, and the generalization of trained skills to real-world settings remains largely unexamined. Whyte et al. (2015), for instance, emphasizes the lack of empirical evidence supporting the transfer of learned social communicative skills to everyday life. With the exception of studies assessing attentional skills—often through comparisons with traditional continuous performance tests (CPTs)—few investigations have directly compared the effectiveness or ecological validity of VR-based interventions with conventional assessment tools.

The majority of studies involving ASD and ADHD clinical populations focus on assessing or training specific target domains without providing a comprehensive evaluation of participants' broader neuropsychological profiles. Consequently, particularly in the case of ASD, assessments often concentrate on isolated social skills while neglecting the potential influence of domain-general cognitive processes, such as executive functions. This narrow focus may limit the interpretation of outcomes and the development of integrated intervention strategies. Furthermore, there is a notable gap in the literature concerning younger children, specifically those aged 3–5 years with suspected ASD. Given its potential for ecological and engaging interaction, VR could play a critical role in both early screening—by assessing core social behaviors such as gaze direction or pointing gestures—and in delivering age-appropriate, sensitive training programs aimed at enhancing early sociocognitive development. Finally, we note a lack of studies targeting minimally verbal or low-IQ participants.

In conclusion, a great variety of VR designs are observed, making it difficult to define which design is more efficient for cognitive and/or social assessment and training in pediatric populations. Accordingly, the clinical design of the majority of studies seems to be restricted to the target population and a specific domain. Future studies should focus on the development of more complex VE in terms of assessment or training (e.g., assessment of more general domains such as EFs and ToM). These VEs should incorporate increasing levels of task complexity and be supported by robust clinical designs, including the use of a control group, comparison with traditional assessment, and an evaluation of transfer effect and the generalization of trained skills to daily-life functioning.
