# Correction: Virtual reality programs targeting executive functions and social cognition evaluation and/or rehabilitation in children with ADHD or ASD—A narrative review

**DOI:** 10.3389/fpsyg.2025.1740118

**Published:** 2025-11-28

**Authors:** Filippia Doulou, Pascale Piolino, Nathalie Angeard

**Affiliations:** Laboratoire Mémoire, Cerveau et Cognition (UR 7536), Institut de Psychologie, Université de Paris Cité, Paris, France

**Keywords:** ADHD, ASD, assessment, neurodevelopmental disorders, pediatric population, training, virtual reality

In the published article, there was an error in affiliation of the three authors. It was erroneously written as:

“Laboratoire Mémoire et Cognition, Université Paris Descartes, Paris, France”,

The correct affiliation appears below:

“Laboratoire Mémoire, Cerveau et Cognition (UR 7536), Institut de Psychologie, Université de Paris Cité, Paris, France.”

The original article has been updated.

