# Supplementary material for: Virtual reality programs targeting executive functions and social cognition evaluation and/or rehabilitation in children with ADHD or ASD—A narrative review
Source: Front Psychol. 2025 Nov 5;16:1583052. doi: 10.3389/fpsyg.2025.1583052 (PMC12626792; doi:10.3389/fpsyg.2025.1583052)
Supplement: Supplementary file 1 [file Data_Sheet_1.docx]

**References**

Abell, F., Happe, F., & Frith, U. (2000). Do triangles play tricks? Attribution of mental states to animated shapes in normal and abnormal development. *Cognitive Development*, *15*(1), 1-16.

Abirached, B., Zhang, Y., Aggarwal, J. K., Tamersoy, B., Fernandes, T., Miranda, J. C., & Orvalho, V. (2011, November). Improving communication skills of children with ASDs through interaction with virtual characters. In *2011 IEEE 1st international conference on serious games and applications for health (SeGAH)* (pp. 1-4). IEEE.

Adams, R., Finn, P., Moes, E., Flannery, K., & Rizzo, A. S. (2009). Distractibility in attention/deficit/hyperactivity disorder (ADHD): The virtual reality classroom. *Child neuropsychology*, *15*(2), 120-135.

Agarwal, R., & Karahanna, E. (2000). Time flies when you're having fun: Cognitive absorption and beliefs about information technology usage. MIS quarterly, 665-694.

Amaral, C. P., Simões, M. A., Mouga, S., Andrade, J., & Castelo-Branco, M. (2017). A novel brain computer interface for classification of social joint attention in autism and comparison of 3 experimental setups: a feasibility study. *Journal of neuroscience methods*, *290*, 105-115.

Anderson, P. (2002). Assessment and development of executive function (EF) during childhood. *Child neuropsychology*, *8*(2), 71-82.

Anderson, V., Spencer-Smith, M., & Wood, A. (2011). Do children really recover better? Neurobehavioural plasticity after early brain insult. *Brain*, *134*(8), 2197-2221.

Arthur, G. (1952). Letter International Performance Scale. Washington, DC: Psychological Service Centre.

Attwood, T. (2004a). James and the maths test. *Exploring feelings: Cognitive behaviour therapy to manage anxiety*, 65-66.

Attwood, T. (2004b). Dylan is being teased. *Exploring feelings: Cognitive behaviour therapy to manage anger*, 65-66.

Baddeley, A. D., & Hitch, G. J. (1974). Working memory. In G. H. Bower (Ed.), *The psychology of learning and motivation Vol. 8* (pp. 47–89). New York: Academic Press.

Barkley, R. A., & Murphy, K. R. (2010). Impairment in occupational functioning and adult ADHD: the predictive utility of executive function (EF) ratings versus EF tests. *Archives of clinical neuropsychology*, *25*(3), 157-173.

Baron-Cohen, S., Wheelwright, S., Hill, J., Raste, Y., & Plumb, I. (2001). The “Reading the Mind in the Eyes” Test revised version: a study with normal adults, and adults with Asperger syndrome or high-functioning autism. *The Journal of Child Psychology and Psychiatry and Allied Disciplines*, *42*(2), 241-251.

Baron-Cohen, S., Leslie, A.M. & Frith, U. (1985). Does the autistic child have a ‘theory of mind’? Cognition, 21(1), 37-46.

Bashiri, A., Ghazisaeedi, M., & Shahmoradi, L. (2017). The opportunities of virtual reality in the rehabilitation of children with attention deficit hyperactivity disorder: a literature review. *Korean journal of pediatrics*, *60*(11), 337.

Bauminger N (2007) Group social-multimodal intervention for HFASD. Journal of Autism and Developmental Disorders 37: 1605–1615.

Bauminger, N., Goren-Bar, D., Gal, E., Weiss, P. L., Yifat, R., Kupersmitt, J., ... & Zancanaro, M. (2007, October). Enhancing social communication in high-functioning children with autism through a co-located interface. In *2007 IEEE 9th Workshop on Multimedia Signal Processing* (pp. 18-21). IEEE.

Bauminger-Zviely, N., Eden, S., Zancanaro, M., Weiss, P. L., & Gal, E. (2013). Increasing social engagement in children with high-functioning autism spectrum disorder using collaborative technologies in the school environment. *Autism*, *17*(3), 317-339.

Beaumont, R., & Sofronoff, K. (2008). A multi‐component social skills intervention for children with Asperger syndrome: The Junior Detective Training Program. *Journal of Child Psychology and Psychiatry*, *49*(7), 743-753.

Bekele, E., Crittendon, J., Zheng, Z., Swanson, A., Weitlauf, A., Warren, Z., & Sarkar, N. (2014). Assessing the utility of a virtual environment for enhancing facial affect recognition in adolescents with autism. *Journal of autism and developmental disorders*, *44*, 1641-1650.

Benzing, V., & Schmidt, M. (2017). Cognitively and physically demanding exergaming to improve executive functions of children with attention deficit hyperactivity disorder: a randomised clinical trial. *BMC pediatrics*, *17*, 1-8.

Benso, F., Chiorri, C., Ardu, E., Venuti, P., & Pasqualotto, A. (2025). Beyond modular and non-modular states: theoretical considerations, exemplifications, and practical implications. *Frontiers in Psychology*, *16*, 1456587.

Benton, A. L., Sivan, A. B., Hamsher, K., Varney, N. R., & Spreen, O. (1983). Contributions to neuropsychological assessment. New York: Oxford University Press.

Bernardini, S., Porayska-Pomsta, K., & Smith, T. J. (2014). ECHOES: An intelligent serious game for fostering social communication in children with autism. *Information Sciences*, *264*, 41-60.

Bernard-Opitz, V., Sriram, N., & Nakhoda-Sapuan, S. (2001). Enhancing social problem solving in children with autism and normal children through computer-assisted instruction. *Journal of autism and developmental disorders*, *31*(4), 377-384.

Berument, S. K., Rutter, M., Lord, C., Pickles, A., & Bailey, A. (1999). Autism screening questionnaire: diagnostic validity. The British Journal of Psychiatry, 175(5), 444-451.

Bioulac, S., Lallemand, S., Rizzo, A., Philip, P., Fabrigoule, C., & Bouvard, M. P. (2012). Impact of time on task on ADHD patient’s performances in a virtual classroom. *European Journal of Paediatric Neurology*, *16*(5), 514-521.

Bleckley, M. K., Durso, F. T., Crutchfield, J. M., Engle, R. W., & Khanna, M. M. (2003). Individual differences in working memory capacity predict visual attention allocation. Psychonomic bulletin & review, 10, 884-889.

Bölte, S., Feineis-Matthews, S., Leber, S., Dierks, T., Hubl, D., & Poustka, F. (2002). The development and evaluation of a computer-based program to test and to teach the recognition of facial affect. *International journal of circumpolar health*, *61*(sup2), 61-68.

Bölte, S., Hubl, D., Feineis-Matthews, S., Prvulovic, D., Dierks, T., & Poustka, F. (2006). Facial affect recognition training in autism: can we animate the fusiform gyrus?. *Behavioral neuroscience*, *120*(1), 211.

Bora, E., & Pantelis, C. (2016). Meta-analysis of social cognition in attention-deficit/hyperactivity disorder (ADHD): comparison with healthy controls and autistic spectrum disorder. *Psychological medicine*, *46*(4), 699-716.

Brickenkamp, R., & Zillmer, E. A. (1998). d2 Test of Attention. Göttingen, Germany: Hogrefe & Huber.

Bul, K. C., Franken, I. H., Van der Oord, S., Kato, P. M., Danckaerts, M., Vreeke, L. J., ... & Maras, A. (2015). Development and user satisfaction of “Plan-It Commander,” a serious game for children with ADHD. *Games for health journal*, *4*(6), 502-512.

Bul, K. C., Kato, P. M., Van der Oord, S., Danckaerts, M., Vreeke, L. J., Willems, A., ... & Maras, A. (2016). Behavioral outcome effects of serious gaming as an adjunct to treatment for children with attention-deficit/hyperactivity disorder: a randomized controlled trial. *Journal of medical Internet research*, *18*(2), e26.

Bul, K. C. M., Doove, L. L., Franken, I. H. A., Van Der Oord, S., Kato, P. M., & Maras, A. (2018). A serious game for children with Attention Deficit Hyperactivity Disorder: Who benefits the most? *PLoS ONE, 13*(3), 1–18.

Canty, A. L., Neumann, D. L., & Shum, D. H. (2017a). Using virtual reality to assess theory of mind subprocesses and error types in early and chronic schizophrenia. *Schizophrenia Research: Cognition*, *10*, 15-19.

Canty, A. L., Neumann, D. L., Fleming, J., & Shum, D. H. (2017b). Evaluation of a newly developed measure of theory of mind: The virtual assessment of mentalising ability. *Neuropsychological Rehabilitation*, *27*(5), 834-870.

Carlson, S. M., Claxton, L. J., & Moses, L. J. (2015). The relation between executive function and theory of mind is more than skin deep. *Journal of Cognition and Development*, *16*(1), 186-197.

Casey, B. J., Cohen, J. D., Jezzard, P., Turner, R., Noll, D. C., Trainor, R. J., et al. (1995). Activation of prefrontal cortex in children during a non-spatial working memory task with functional MRI. 2, 221-229.

Castellanos, F. X., Sonuga-Barke, E. J., Milham, M. P., & Tannock, R. (2006). Characterizing cognition in ADHD: beyond executive dysfunction. *Trends in cognitive sciences*, *10*(3), 117-

123.

Cepeda, N. J., Cepeda, M. L., & Kramer, A. F. (2000). Task switching and attention deficit hyperactivity disorder. Journal of Abnormal Child Psychology, 28, 213-226.

Chen, M. T., Chang, Y. P., Marraccini, M. E., Cho, M. C., & Guo, N. W. (2022). Comprehensive attention training system (CATS): A computerized executive-functioning training for school-aged children with autism spectrum disorder. *International Journal of Developmental Disabilities*, *68*(4), 528-537.

Chen, Y., Zhou, Z., Cao, M., Liu, M., Lin, Z., Yang, W., ... & Xiong, P. (2022). Extended Reality (XR) and telehealth interventions for children or adolescents with autism spectrum disorder: Systematic review of qualitative and quantitative studies. *Neuroscience & Biobehavioral Reviews*, 104683.

Cheng, Y., & Chen, S. (2010). Improving social understanding of individuals of intellectual and developmental disabilities through a 3D-facail expression intervention program. *Research in developmental disabilities*, *31*(6), 1434-1442.

Cheng, Y., & Huang, R. (2012). Using virtual reality environment to improve joint attention associated with pervasive developmental disorder. *Research in developmental disabilities*, *33*(6), 2141-2152.

Cho, B. H., Ku, J., Jang, D. P., Kim, S., Lee, Y. H., Kim, I. Y., ... & Kim, S. I. (2002). The effect of virtual reality cognitive training for attention enhancement. *CyberPsychology & Behavior*, *5*(2), 129-137.

Cho, B. H., Kim, S., Shin, D. I., Lee, J. H., Min Lee, S., Young Kim, I., & Kim, S. I. (2004). Neurofeedback training with virtual reality for inattention and impulsiveness. *Cyberpsychology & Behavior*, *7*(5), 519-526.

Cibrian, F. L., Lakes, K. D., Schuck, S. E., & Hayes, G. R. (2022). The potential for emerging technologies to support self-regulation in children with ADHD: A literature review. *International Journal of Child-Computer Interaction*, *31*, 100421.

Ciesielski, K. T., & Harris, R. J. (1997). Factors related to performance failure on executive tasks in autism. *Child Neuropsychology*, *3*(1), 1-12.

Cobb, S., Parsons, S., Millen, L., Eastgate, R., & Glover, T. (2010). Design and development of collaborative technology for children with autism: COSPATIAL. In *INTED2010 Proceedings* (pp. 4374-4383). IATED.

Conners, K. (2010). Conners (3rd ed.). North Tonawanda, NY: Multihealth Systems.

Constantino, JN.; Gruber, CP. The Social Responsiveness Scale. Los Angeles, CA: Western Psychological Services; 2005.

Constantino, J. & Gruber, C. (2002). Social Responsiveness Scale. Los Angeles, CA: Western Psychological Services.

Constantino, J. N., Davis, S. A., Todd, R. D., Schindler, M. K., Gross, M. M., Brophy, S. L., ... & Reich, W. (2003). Validation of a brief quantitative measure of autistic traits: comparison of the social responsiveness scale with the autism diagnostic interview-revised. *Journal of autism and developmental disorders*, *33*(4), 427-433.

Constantino, J. The social responsiveness scale. LA: Western Psychological Services; 2004.

Corcoran, R. (2000). Theory of mind in other clinical conditions: Is a selective ‘theory of mind’deficit exclusive to autism. *Understanding other minds: Perspectives from developmental cognitive neuroscience*, 391-421.

Corsello, C., Hus, V., Pickles, A., Risi, S., Cook Jr, E. H., Leventhal, B. L., & Lord, C. (2007). Between a ROC and a hard place: Decision making and making decisions about using the SCQ. *Journal of child psychology and psychiatry*, *48*(9), 932-940.

Corsi, P. M. (1972). Human memory and the medial temporal region of the brain.

De Luca, R., Russo, M., Naro, A., Tomasello, P., Leonardi, S., Santamaria, F., ... & Calabrò, R. S. (2018). Effects of virtual reality-based training with BTs-Nirvana on functional recovery in stroke patients: preliminary considerations. *International Journal of Neuroscience*, *128*(9), 791-796.

Dennis, M., Spiegler, B. J., Simic, N., Sinopoli, K. J., Wilkinson, A., Yeates, K. O., ... & Fletcher, J. M. (2014). Functional plasticity in childhood brain disorders: when, what, how, and whom to assess. *Neuropsychology review*, *24*(4), 389-408.

Deriso, D., Susskind, J., Krieger, L., & Bartlett, M. (2012). Emotion mirror: a novel intervention for autism based on real-time expression recognition. In *Computer Vision–ECCV 2012. Workshops and Demonstrations: Florence, Italy, October 7-13, 2012, Proceedings, Part III 12* (pp. 671-674). Springer Berlin Heidelberg.

Devine, R. T., & Hughes, C. (2014). Relations between false belief understanding and executive function in early childhood: A meta‐analysis. *Child development*, *85*(5), 1777-1794.

De Jonge, M., & De Bildt, A. (2007). Nederlandse bewerking van de ADI-R. Amsterdam: Hogrefe Uitgevers BV.

De Vries, M., & Geurts, H. M. (2012). Cognitive flexibility in ASD; task switching with emotional faces. Journal of autism and developmental disorders, 42, 2558-2568.

de Vries, M., Prins, P. J., Schmand, B. A., & Geurts, H. M. (2015). Working memory and cognitive flexibility‐training for children with an autism spectrum disorder: A randomized controlled trial. *Journal of Child Psychology and Psychiatry*, *56*(5), 566-576.

Diamond, A. (2013). Executive functions. *Annual review of psychology*, *64*, 135.

Diamond, A., & Ling, D. S. (2020). Review of the evidence on, and fundamental questions about, efforts to improve executive functions, including working memory.

Didehbani, N., Allen, T., Kandalaft, M., Krawczyk, D., & Chapman, S. (2016). Virtual reality social cognition training for children with high functioning autism. *Computers in human behavior*, *62*, 703-711.

Dobrean, A., Raven, J., Comşa, M., Rusu, C., & Balazsi, R. (2008). The Romanian standardisation of the standard progressive matrices plus: Sample and general results. Uses and abuses of intelligence: Studies advancing Spearman and Raven’s quest for non arbitrary metrics. Cluj-Napoca, Romania: Romanian Testing Services.

Domuța, A., Balazsi, R., Comșa, M., & Rusu, C. (2004). Standardizarea pe populaţia României a testului Matrici Progresive Raven Standard Plus. *Psihologia resurselor umane*, *2*(1), 50-56.

Dovis, S., Van der Oord, S., Wiers, R. W., & Prins, P. J. (2015). Improving executive functioning in children with ADHD: training multiple executive functions within the context of a computer game. A randomized double-blind placebo controlled trial. *PloS one*, *10*(4), e0121651.

Dunn, L. M., Dunn, L. M., Whetton, C., & Pintilie, D. (1981). British Picture Vocabulary Test. CITY: NFER-Nelson.

Dunn, L. M., Dunn, L. M., Whetton, C. & Pintilie, D. (1982). British Picture Vocabulary Scale. London: NFER-Nelson

Dunn LM and Dunn LM (1997) *Examiner’s Manual for the Peabody Picture Vocabulary Test*. 3rd ed. Circle Pines, MN: American Guidance Service.

Dunn, L. M., Dunn, D. M., Styles, B. & NFER - Sewell, J. (2009). The British Picture Vocabulary Scale 3rd Edition (BPVS III). GL Assessment, London, UK.

Duval, C., Desgranges, B., Eustache, F., & Piolino, P. (2009). Le soi à la loupe des neurosciences cognitives. *Psychologie & NeuroPsychiatrie du vieillissement*, *7*(1), 7-19.

Ehlers, S., Gillberg, C., & Wing, L. (1999). A screening questionnaire for Asperger syndrome and other high-functioning autism spectrum disorders in school age children. Journal of autism and developmental disorders, 29, 129-141.

Elliott, C. D. (2007). Differential Ability Scales—Second Edition (DAS-II) San Antonio. *TX: Harcourt Assessment*.

Emslie, H., Wilson, C. F., Burden, V., Nimmo-Smith, I., Wilson, B. A. (2003). Behavioural Assessment of the Dysexecutive Syndrome for Children (BADS-C), Thames Valley Test Company, Bury St. Edmunds.

Escobedo, L., Nguyen, D. H., Boyd, L., Hirano, S., Rangel, A., Garcia-Rosas, D., ... & Hayes, G. (2012, May). MOSOCO: a mobile assistive tool to support children with autism practicing social skills in real-life situations. In *Proceedings of the SIGCHI conference on human factors in computing systems* (pp. 2589-2598).

Faja, S., Aylward, E., Bernier, R., & Dawson, G. (2007). Becoming a face expert: A computerized face-training program for high-functioning individuals with autism spectrum disorders. *Developmental neuropsychology*, *33*(1), 1-24.

Farré, A., & Narbona, J. (2003). EDAH. evaluación del trastorno por déficit de atención con hiperactividad. Madrid, Spain: TEA.

Fernandes, T., Alves, S., Miranda, J., Queirós, C., & Orvalho, V. (2011). LIFEisGAME: A facial character animation system to help recognize facial expressions. In *ENTERprise Information Systems: International Conference, CENTERIS 2011, Vilamoura, Portugal, October 5-7, 2011, Proceedings, Part III* (pp. 423-432). Springer Berlin Heidelberg.

Fernández-Sotos, P., Fernández-Caballero, A., & Rodriguez-Jimenez, R. (2020). Virtual reality for psychosocial remediation in schizophrenia: a systematic review. *The European Journal of Psychiatry*, *34*(1), 1-10.

Fiske, A., & Holmboe, K. (2019). Neural substrates of early executive function development. Developmental Review, 52, 42-62.

Fletcher-Watson, S., Hammond, S. T., O'Hare, A., Pain, H., Petrou, A., & McConachie, H. (2013, May). Click-east: evaluating the impact of an ipad app on social communicative abilities in young children with autism. In *International meeting for autism research*.

Fletcher‐Watson, S., McConnell, F., Manola, E., & McConachie, H. (2014). Interventions based on the Theory of Mind cognitive model for autism spectrum disorder (ASD). *Cochrane Database of Systematic Reviews*, (3).

Fletcher-Watson, S., Petrou, A., Scott-Barrett, J., Dicks, P., Graham, C., O’Hare, A., et al. (2016). A trial of an iPad TM intervention targeting social communication skills in children with autism. Autism, 20, 771–782. http://dx.doi.org/10. 1177/1362361315605624, LK.

Friedman, N. P., & Robbins, T. W. (2022). The role of prefrontal cortex in cognitive control and executive function. *Neuropsychopharmacology*, *47*(1), 72-89.

Frith, C. D., & Frith, U. (2006). The neural basis of mentalizing. *Neuron*, *50*(4), 531-534.

Frith, C. D., & Frith, U. (2007). Social cognition in humans. *Current biology*, *17*(16), R724-R732.

Frolli, A., Savarese, G., Di Carmine, F., Bosco, A., Saviano, E., Rega, A., ... & Ricci, M. C. (2022). Children on the autism spectrum and the use of virtual reality for supporting social skills. *Children*, *9*(2), 181.

Fuchs, P., Moreau, G., & Guitton, P. (Eds.). (2011). Virtual reality: concepts and technologies. CRC Press.

Gallagher, H. L., & Frith, C. D. (2003). Functional imaging of ‘theory of mind’. *Trends in cognitive sciences*, *7*(2), 77-83.

Garfield, J. L., Peterson, C. C., & Perry, T. (2001). Social cognition, language acquisition and the development of the theory of mind. Mind & Language, 16(5), 494-541.

Ghanouni, P., Jarus, T., Zwicker, J. G., Lucyshyn, J., Mow, K., & Ledingham, A. (2019). Social stories for children with autism spectrum disorder: Validating the content of a virtual reality program. *Journal of autism and developmental disorders*, *49*(2), 660-668.

Gioia, G. A., Isquith, P. K., Guy, S. C., & Kenworthy, L. (2000). Test review behavior rating inventory of executive function. Child Neuropsychology, 6(3), 235-238.

Golan, O. (2006). Systemising emotions: Teaching emotion recognition to people with autism using interactive multimedia. Unpublished doctoral dissertation, University of Cambridge, Cambridge, UK.

Gorisse, G., Christmann, O., Amato, E. A., & Richir, S. (2017). First-and third-person perspectives in immersive virtual environments: presence and performance analysis of embodied users. *Frontiers in Robotics and AI*, *4*, 33.

Gordon, I., Pierce, M. D., Bartlett, M. S., & Tanaka, J. W. (2014). Training facial expression production in children on the autism spectrum. *Journal of autism and developmental disorders*, *44*, 2486-2498.

Grossard, C., Grynspan, O., Serret, S., Jouen, A. L., Bailly, K., & Cohen, D. (2017). Serious games to teach social interactions and emotions to individuals with autism spectrum disorders (ASD). *Computers & Education*, *113*, 195-211.

Grynszpan, O., Martin, J. C., & Nadel, J. (2008). Multimedia interfaces for users with high functioning autism: An empirical investigation. *International Journal of Human-Computer Studies*, *66*(8), 628-639.

Grynszpan, O., Nadel, J., Martin, J. C., Simonin, J., Bailleul, P., Wang, Y., ... & Constant, J. (2012). Self-monitoring of gaze in high functioning autism. *Journal of autism and developmental disorders*, *42*(8), 1642-1650.

Gualtieri, C. T., & Johnson, L. G. (2005). ADHD: Is objective diagnosis possible?. *Psychiatry (Edgmont)*, *2*(11), 44.

Gutiérrez-Maldonado, J., Letosa-Porta, À., Rus-Calafell, M., & Peñaloza-Salazar, C. (2009). The assessment of attention deficit hyperactivity disorder in children using continous performance tasks in virtual environments. *Anuario de Psicología*, *40*(2), 211-222.

Hahs, A. D. (2015). Teaching prerequisite perspective-taking skills to children with autism. *International Journal of Psychology and Behavioral Sciences*, *5*(3), 115-120.

Hartman, C. A., Luteijn, E., Moorlag, A., De Bildt, A., & Minderaa, R. (2007). Manual for the CSBQ [Handleiding voor de VISK].

Happé, F. G. (1994). An advanced test of theory of mind: Understanding of story characters' thoughts and feelings by able autistic, mentally handicapped, and normal children and adults. Journal of autism and Developmental disorders, 24(2), 129-154.

Herrera, G., Alcantud, F., Jordan, R., Blanquer, A., Labajo, G., & De Pablo, C. (2008). Development of symbolic play through the use of virtual reality tools in children with autistic spectrum disorders: Two case studies. *Autism*, *12*(2), 143-157.

Hofmann, S. G., Doan, S. N., Sprung, M., Wilson, A., Ebesutani, C., Andrews, L. A., ... & Harris, P. L. (2016). Training children’s theory-of-mind: A meta-analysis of controlled studies. *Cognition*, *150*, 200-212.

Hopkins, I. M., Gower, M. W., Perez, T. A., Smith, D. S., Amthor, F. R., Casey Wimsatt, F., & Biasini, F. J. (2011). Avatar assistant: improving social skills in students with an ASD through a computer-based intervention. *Journal of autism and developmental disorders*, *41*(11), 1543-1555.

Howard, M. C., & Gutworth, M. B. (2020). A meta-analysis of virtual reality training programs for social skill development. *Computers & Education*, *144*, 103707.

Howlin, P., Baron-Cohen, S., & Hadwin, J. A. (1999). *Teaching children with autism to mind-read: A practical guide for teachers and parents*. John Wiley & Sons.

Ip, H. H., Wong, S. W., Chan, D. F., Byrne, J., Li, C., Yuan, V. S., ... & Wong, J. Y. (2018). Enhance emotional and social adaptation skills for children with autism spectrum disorder: A virtual reality enabled approach. *Computers & Education*, *117*, 1-15.

Irish, J. E. (2013). Can I sit here? A review of the literature supporting the use of single-user virtual environments to help adolescents with autism learn appropriate social communication skills. *Computers in Human Behavior*, *29*(5), A17-A24.

Iriye, H., & St Jacques, P. L. (2021). Memories for third-person experiences in immersive virtual reality. *Scientific reports*, *11*(1), 1-14.

Jolles, D. D., & Crone, E. A. (2012). Training the developing brain: a neurocognitive perspective. *Frontiers in human neuroscience*, *6*, 76.

Jung, K. E., Lee, H. J., Lee, Y. S., Cheong, S. S., Choi, M. Y., Suh, D. S., ... & Lee, J. H. (2006). The Application of a Sensory Integration Treatment Based on Virtual Reality-Tangible Interaction for Children with Autistic Spectrum Disorder. *PsychNology J.*, *4*(2), 145-159.

Kamphaus, R. W., & Reynolds, C. R. (1998). BASC monitor for ADHD. *Circle Pines, MN: AGS Publishing*.

Kandalaft, M. R., Didehbani, N., Krawczyk, D. C., Allen, T. T., & Chapman, S. B. (2013). Virtual reality social cognition training for young adults with high-functioning autism. *Journal of autism and developmental disorders*, *43*(1), 34-44.

Kaplan-Rakowski, R., & Gruber, A. (2019). Low-immersion versus high-immersion virtual reality: Definitions, classification, and examples with a foreign language focus. In *Proceedings of the Innovation in Language Learning International Conference 2019* (pp. 552-555).

Karmiloff-Smith, A. (2018). An alternative to domain-general or domain-specific frameworks for theorizing about human evolution and ontogenesis. In *Thinking developmentally from constructivism to neuroconstructivism* (pp. 289-304). Routledge.

Kaufman, A. S., & Kaufman, N. L. (1990). Kaufman Brief Intelligence Test. Circle Pines, MN: American Guidance Service.

Ke, F., & Im, T. (2013). Virtual-reality-based social interaction training for children with high-functioning autism. *The Journal of Educational Research*, *106*(6), 441-461.

Ke, F., Moon, J., & Sokolikj, Z. (2022). Virtual reality–based social skills training for children with autism spectrum disorder. *Journal of Special Education Technology*, *37*(1), 49-62.

Kennedy, R. S., Lane, N. E., Berbaum, K. S., & Lilienthal, M. G. (1993). Simulator sickness questionnaire: An enhanced method for quantifying simulator sickness. *The international journal of aviation psychology*, *3*(3), 203-220.

Kilteni, K., Groten, R., & Slater, M. (2012). The sense of embodiment in virtual reality. *Presence: Teleoperators and Virtual Environments*, *21*(4), 373-387.

Kim, K., Geiger, P., Herr, NR., Rosenthal, MZ. The virtual reality emotion sensitivity test (V-REST): Development and construct validity. Presented at the Association for Behavioral and Cognitive Therapies (ABCT) conference; November 18–21, 2010; San Francisco, CA. 2010.

Kim, K., Rosenthal, M. Z., Gwaltney, M., Jarrold, W., Hatt, N., McIntyre, N., ... & Mundy, P. (2015). A virtual joy-stick study of emotional responses and social motivation in children with autism spectrum disorder. *Journal of autism and developmental disorders*, *45*(12), 3891-3899.

Kloo, D., & Perner, J. (2008). Training theory of mind and executive control: A tool for improving school achievement?. *Mind, Brain, and Education*, *2*(3), 122-127.

Korkman, M., Kirk, U., & Kemp, S. (2007). NEPSY-II. San Antonio, TX: Harcourt Assessment, Inc.

Kort, D. W., Compaan, E. L., Bleichrodt, N., Resing, W. C. M., Schittekatte, M., Bosmans, M., & Verhaeghe, P. (2002). WISC-III nl handleiding. Dutch Manual). Amsterdam: NIP.

Krasny-Pacini, A., Limond, J., & Chevignard, M. (2016). Rééducation des fonctions exécutives chez l’enfant cérébro-lésé. *Approche neuropsychologique des apprentissages chez l’enfant*, *28*(141), 185-197.

Krug, D. A., Arick, J. R., & Almond, P. G. (1979). Autism screening instrument for educational planning: Background and development. In J. Gilliam (Ed.), Autism: Diagnosis, instruction, management and research. Austin, TX: University of Texas Press.

Lacava, P. G., Golan, O., Baron-Cohen, S., & Smith Myles, B. (2007). Using assistive technology to teach emotion recognition to students with Asperger syndrome: A pilot study. *Remedial and Special Education*, *28*(3), 174-181.

Lahiri, U., Bekele, E., Dohrmann, E., Warren, Z., & Sarkar, N. (2012). Design of a virtual reality based adaptive response technology for children with autism. *IEEE Transactions on Neural Systems and Rehabilitation Engineering*, *21*(1), 55-64.

Lakes, K. D., Cibrian, F. L., Schuck, S., Nelson, M., & Hayes, G. R. (2022). Digital health interventions for youth with ADHD: A systematic review. *Computers in Human Behavior Reports*, 100174.

Le Couteur, A., Rutter, M., Lord, C., Rios, P., Robertson, S., Holdgrafer, M., & McLennan, J. (1989). Autism diagnostic interview: a standardized investigator-based instrument. *Journal of autism and developmental disorders*, *19*, 363-387.

Lee, J. M., Cho, B. H., Ku, J. H., Kim, J. S., Lee, J. H., Kim, I. Y., & Kim, S. I. (2001, October). A study on the system for treatment of ADHD using virtual reality. In *2001 Conference Proceedings of the 23rd Annual International Conference of the IEEE Engineering in Medicine and Biology Society* (Vol. 4, pp. 3754-3757). IEEE.

Lee, K. M. (2004). Presence, explicated. *Communication theory*, *14*(1), 27-50.

Lee, K., Bull, R., & Ho, R. M. (2013). Developmental changes in executive functioning. *Child development*, *84*(6), 1933-1953.

Lenormand, D., & Piolino, P. (2022). In search of a naturalistic neuroimaging approach: exploration of general feasibility through the case of VR-fMRI and application in the domain of episodic memory. *Neuroscience & Biobehavioral Reviews*, *133*, 104499.

Liu, R., Salisbury, J. P., Vahabzadeh, A., & Sahin, N. T. (2017). Feasibility of an autism-focused augmented reality smartglasses system for social communication and behavioral coaching. *Frontiers in pediatrics*, *5*, 145.

Logan, G. D. (1994). In Carr T. H., Dagenbach D. (Eds.), On the ability to inhibit thought and action: A users' guide to the stop signal paradigm. San Diego: Academic Press

Loh, H. W., Ooi, C. P., Barua, P. D., Palmer, E. E., Molinari, F., & Acharya, U. (2022). Automated detection of ADHD: Current trends and future perspective. *Computers in Biology and Medicine*, 105525.

Loomis, J. M., Blascovich, J. J., & Beall, A. C. (1999). Immersive virtual environment technology as a basic research tool in psychology. Behavior research methods, instruments, & computers, 31(4), 557-564.

Lord C, Rutter M, Le Couteur A. Autism Diagnostic Interview-Revised: a revised version of a diagnostic interview for caregivers of individuals with possible pervasive developmental disorders. J Autism Dev Disord 1994; 24: 659-685.

Lord C, Risi S, Lambrecht L, et al. (2000) The autism diagnostic observation schedule-generic: a standard measure of social and communication deficits associated with the spectrum of autism. *Journal of Autism and Developmental Disorders* 30(3): 205–223.

Lord, C., Rutter, M., DiLavore, P., & Risi, S. (2001). Autism Diagnostic Observation Schedule (ADOS). Los Angeles: Western Psychological Services.

Lorenzo, G., Lledó, A., Pomares, J., & Roig, R. (2016). Design and application of an immersive virtual reality system to enhance emotional skills for children with autism spectrum disorders. *Computers & Education*, *98*, 192-205.

Maggio, M. G., De Luca, R., Molonia, F., Porcari, B., Destro, M., Casella, C., ... & Calabro, R. S. (2019). Cognitive rehabilitation in patients with traumatic brain injury: A narrative review on the emerging use of virtual reality. *Journal of Clinical Neuroscience*, *61*, 1-4.

March, J. S. (1997). Multidimensional anxiety scale for children.

Mazon, C., Fage, C., & Sauzéon, H. (2019). Effectiveness and usability of technology-based interventions for children and adolescents with ASD: A systematic review of reliability, consistency, generalization and durability related to the effects of intervention. *Computers in Human Behavior*, *93*, 235-251.

Minzenberg, M. J., Laird, A. R., Thelen, S., Carter, C. S., & Glahn, D. C. (2009). Meta-analysis of 41 functional neuroimaging studies of executive function in schizophrenia. *Archives of general psychiatry*, *66*(8), 811-822.

Mitchell, P., Parsons, S., & Leonard, A. (2007). Using virtual environments for teaching social understanding to 6 adolescents with autistic spectrum disorders. *Journal of autism and developmental disorders*, *37*(3), 589-600.

Moore, D., Cheng, Y., McGrath, P., & Powell, N. J. (2005). Collaborative virtual environment technology for people with autism. *Focus on autism and other developmental disabilities*, *20*(4), 231-243.

Mühlberger, A., Jekel, K., Probst, T., Schecklmann, M., Conzelmann, A., Andreatta, M., ... & Romanos, M. (2020). The influence of methylphenidate on hyperactivity and attention deficits in children with ADHD: a virtual classroom test. *Journal of attention disorders*, *24*(2), 277-289.

Müller, U., & Kerns, K. (2015). The development of executive function.

Mundy, P., Kim, K., McIntyre, N., Lerro, L., & Jarrold, W. (2016). Brief report: Joint attention and information processing in children with higher functioning autism spectrum disorders. *Journal of autism and developmental disorders*, *46*(7), 2555-2560.

Myles, B. S., Bock, S. J., & Simpson, R. L. (2001). Asperger syndrome di- agnostic scale. Austin, TX: PRO-ED.

Neguţ, A., Matu, S. A., Sava, F. A., & David, D. (2016). Task difficulty of virtual reality-based assessment tools compared to classical paper-and-pencil or computerized measures: A meta-analytic approach. *Computers in Human Behavior*, *54*, 414-424.

Neguț, A., Jurma, A. M., & David, D. (2017). Virtual-reality-based attention assessment of ADHD: ClinicaVR: Classroom-CPT versus a traditional continuous performance test. *Child Neuropsychology*, *23*(6), 692-712.

Niendam, T. A., Laird, A. R., Ray, K. L., Dean, Y. M., Glahn, D. C., & Carter, C. S. (2012). Meta-analytic evidence for a superordinate cognitive control network subserving diverse executive functions. *Cognitive, Affective, & Behavioral Neuroscience*, *12*(2), 241-268.

Nolin, P., Stipanicic, A., Henry, M., Lachapelle, Y., Lussier-Desrochers, D., & Allain, P. (2016). ClinicaVR: Classroom-CPT: A virtual reality tool for assessing attention and inhibition in children and adolescents. *Computers in Human Behavior*, *59*, 327-333.

Norman, D. A., & Shallice, T. (1986). Attention to action: Willed and automatic control of behavior. In *Consciousness and self-regulation: Advances in research and theory volume 4* (pp. 1-18). Boston, MA: Springer US.

O'Hearn, K., Asato, M., Ordaz, S., & Luna, B. (2008). Neurodevelopment and executive function in autism. *Development and psychopathology*, *20*(4), 1103-1132.

Oosterlaan, J., Scheres, A., Antrop, I., Roeyers, H., & Sergeant, J. A. (2000). Handleiding bij de vragenlijst voor gedragsproblemen bij kinderen VvGK.

Parenti, I., Rabaneda, L. G., Schoen, H., & Novarino, G. (2020). Neurodevelopmental disorders: from genetics to functional pathways. *Trends in Neurosciences*, *43*(8), 608-621.

Parsons, S., & Mitchell, P. (2002). The potential of virtual reality in social skills training for people with autistic spectrum disorders. *Journal of intellectual disability research*, *46*(5), 430-443.

Parsons, S., Mitchell, P., & Leonard, A. (2004). The use and understanding of virtual environments by adolescents with autistic spectrum disorders. *Journal of Autism and Developmental disorders*, *34*(4), 449-466.

Parsons, S., Leonard, A., & Mitchell, P. (2006). Virtual environments for social skills training: comments from two adolescents with autistic spectrum disorder. *Computers & Education*, *47*(2), 186-206.

Parsons, T. D., Bowerly, T., Buckwalter, J. G., & Rizzo, A. A. (2007). A controlled clinical comparison of attention performance in children with ADHD in a virtual reality classroom compared to standard neuropsychological methods. *Child neuropsychology*, *13*(4), 363-381.

Parsons, S. (2015). Learning to work together: Designing a multi-user virtual reality game for social collaboration and perspective-taking for children with autism. *International Journal of Child-Computer Interaction*, *6*, 28-38.

Pelham Jr, W. E., Gnagy, E. M., Greenslade, K. E., & Milich, R. (1992). Teacher ratings of DSM-III-R symptoms for the disruptive behavior disorders. *Journal of the American Academy of Child & Adolescent Psychiatry*, *31*(2), 210-218.

Pellicano, E. (2007). Links between theory of mind and executive function in young children with autism: clues to developmental primacy. *Developmental psychology*, *43*(4), 974.

Pellicano, E. (2012). The development of executive function in autism. *Autism research and treatment*, *2012*.

Perner, J. & Wimmer, H. (1985). "John thinks that Mary thinks that..." Attribution of second-order beliefs by 5-10 year old children. Journal of Experimental Child Psychology, 39, 437-471.

Peterson, C. C., & Wellman, H. M. (2019). Longitudinal theory of mind (ToM) development from preschool to adolescence with and without ToM delay. *Child Development*, *90*(6), 1917-1934.

Pollak, Y., Shomaly, H. B., Weiss, P. L., Rizzo, A. A., & Gross-Tsur, V. (2010). Methylphenidate effect in children with ADHD can be measured by an ecologically valid continuous performance test embedded in virtual reality. *CNS spectrums*, *15*(2), 125-130.

Posserud, M. B., Lundervold, A. J., & Gillberg, C. (2006). Autistic features in a total population of 7–9‐year‐old children assessed by the ASSQ (Autism Spectrum Screening Questionnaire). Journal of child psychology and psychiatry, 47(2), 167-175.

Prins, P. J., Brink, E. T., Dovis, S., Ponsioen, A., Geurts, H. M., De Vries, M., & Van Der Oord, S. (2013). “Braingame Brian”: toward an executive function training program with game elements for children with ADHD and cognitive control problems. GAMES FOR HEALTH: Research, Development, and Clinical Applications, 2(1), 44-49.

Pugliese, C. E., Anthony, L., Strang, J. F., Dudley, K., Wallace, G. L., & Kenworthy, L. (2015). Increasing adaptive behavior skill deficits from childhood to adolescence in autism spectrum disorder: Role of executive function. *Journal of autism and developmental disorders*, *45*(6), 1579-1587.

Rajendran, G. (1999). *Helping adults with Asperger's syndrome acquire interpersonal understanding: the bubble dialogue computer program* (Doctoral dissertation, University of Birmingham).

Rajendran, G., & Mitchell, P. (2000). Computer mediated interaction in Asperger’s syndrome: The Bubble Dialogue program. *Computers & Education*, *35*(3), 189-207.

Raven, J. C., & Court, J. H. (1986). Raven's progressive matrices and Raven's coloured matrices. *London: HK Lewis*.

Raven, J., & Raven, J. C. (1998). Court, jh (1998). Manual for Raven’s progressive matrices and vocabulary scales.

Ravindran, V., Osgood, M., Sazawal, V., Solorzano, R., & Turnacioglu, S. (2019). Virtual reality support for joint attention using the Floreo Joint Attention Module: Usability and feasibility pilot study. *JMIR pediatrics and parenting*, *2*(2), e14429.

Reynolds, CR., Kamphaus, RW. BASC-2: Behavior assessment system for children, second edition manual. Circle Pines, MN: American Guidance Service; 2004.

Reitan, R. (1992). Trail making test manual for administration and scoring. Tucson, AZ: Reitan Neuropsychology Laboratory.

Reitan, R. M. (1971). Trail making test results for normal and brain-damaged children. Perceptual and Motor Skills, 33, 575–581.

Reitan, R. M., & Wolfson, D. (2004). The trail making test as an initial screening procedure for neuropsychological impairment in older children. Archives of Clinical Neuropsychology, 19, 281–288

Rice, L. M., Wall, C. A., Fogel, A., & Shic, F. (2015). Computer-assisted face processing instruction improves emotion recognition, mentalizing, and social skills in students with asd. Journal of autism and developmental disorders, 45(7), 2176-2186.

Rizzo, A. A., & Buckwalter, J. G. (1997). Virtual reality and cognitive assessment. *Virtual reality in neuro-psycho-physiology: Cognitive, clinical and methodological issues in assessment and rehabilitation*, *44*, 123.

Rizzo, A. A., Buckwalter, J. G., Bowerly, T., Van Der Zaag, C., Humphrey, L., Neumann, U., ... & Sisemore, D. (2000). The virtual classroom: a virtual reality environment for the assessment and rehabilitation of attention deficits. *CyberPsychology & Behavior*, *3*(3), 483-499.

Rizzo, A. A., Bowerly, T., Buckwalter, J. G., Klimchuk, D., Mitura, R., & Parsons, T. D. (2009). A virtual reality scenario for all seasons: the virtual classroom. *Cns Spectrums*, *11*(1), 35-44.

Robertson, I. H., Manly, T., Andrade, J., Baddeley, B. T., & Yiend, J. (1997). Oops!': performance correlates of everyday attentional failures in traumatic brain injured and normal subjects. *Neuropsychologia*, *35*(6), 747-758.

Robinson, S., Goddard, L., Dritschel, B., Wisley, M., & Howlin, P. (2009). Executive functions in children with autism spectrum disorders. *Brain and cognition*, *71*(3), 362-368.

Rodríguez, C., Areces, D., García, T., Cueli, M., & González-Castro, P. (2018). Comparison between two continuous performance tests for identifying ADHD: Traditional vs. virtual reality. *International journal of clinical and health psychology*, *18*(3), 254-263.

Roeyers, H., Thys, M., Druart, C., De Schryver, M., & Schittekatte, M. (2011). SRS, Screeningslijst voor autismespectrumstoornissen.

Rold, G. Stanford-Binet Intelligence Scales. 5. Rolling Meadows, IL: Riverside; 2003. Nelson Education

Rose, T., Nam, C. S., & Chen, K. B. (2018). Immersion of virtual reality for rehabilitation-Review. Applied ergonomics, 69, 153-161.

Rutter M, Bailey A, Berument SK, et al. (2003) *Social Communication Questionnaire*. Los Angeles, CA: Western Psychological Services.

Sanchez-Vives, M. V., & Slater, M. (2005). From presence to consciousness through virtual reality. Nature reviews neuroscience, 6(4), 332-339.

Sattler, J. M. (2001). *Assessment of children: Cognitive applications*. Jerome M Sattler Publisher.

Schöne, B., Wessels, M., & Gruber, T. (2019). Experiences in virtual reality: A window to autobiographical memory. *Current Psychology*, *38*(3), 715-719.

Schopler, E., Reichler, R. J., DeVellis, R. F., & Daly, K. (1980). Toward objective classification of childhood autism: Childhood Autism Rating Scale (CARS). Journal of autism and developmental disorders.

Schopler, E., Lansing, M. D., Reichler, R. J., & Marcus, L. M. (2004). Psychoeducational profile third edition (PEP-3). *Pro-ed, USA*.

Scott, F. J., Baron-Cohen, S., Bolton, P., & Brayne, C. (2002). The CAST (Childhood Asperger Syndrome Test) Preliminary development of a UK screen for mainstream primary-school-age children. *Autism*, *6*(1), 9-31.

Serret, S., Hun, S., Iakimova, G., Lozada, J., Anastassova, M., Santos, A., ... & Askenazy, F. (2014). Facing the challenge of teaching emotions to individuals with low-and high-functioning autism using a new serious game: a pilot study. *Molecular autism*, *5*(1), 1-17.

Shaffer, D., Fisher, P., Lucas, C. P., Dulcan, M. K., & Schwab-Stone, M. E. (2000). NIMH Diagnostic Interview Schedule for Children Version IV (NIMH DISC-IV): description, differences from previous versions, and reliability of some common diagnoses. Journal of the American Academy of Child & Adolescent Psychiatry, 39(1), 28-38.

Shamay-Tsoory, S. G., Tibi-Elhanany, Y., & Aharon-Peretz, J. (2006). The ventromedial prefrontal cortex is involved in understanding affective but not cognitive theory of mind stories. *Social neuroscience*, *1*(3-4), 149-166.

Sheslow, D., & Adams, W. (2003). Wide-range assessment of memory and learning (2nd ed.). Lutz, FL: Psychological Assessment Resources.

Silver, M., & Oakes, P. (2001). Evaluation of a new computer intervention to teach people with autism or Asperger syndrome to recognize and predict emotions in others. *Autism*, *5*(3), 299-316.

Skalski, S., Konaszewski, K., Pochwatko, G., Balas, R., & Surzykiewicz, J. (2021). Effects of hemoencephalographic biofeedback with virtual reality on selected aspects of attention in children with ADHD. *International Journal of Psychophysiology*, *170*, 59-66.

Smidts, D. P., & Huizinga, M. (2010). BRIEF executieve functies gedragsvragenlijst: Handleiding.

Smith, E. E., & Jonides, J. (1999). Storage and executive processes in the frontal lobes. Science, 283(5408), 1657-1661.

Sparrow, S., Cicchetti, D., & Balla, D. (2005). Vineland adaptive behavior scales second edition. Circle Pines, MN: American Guidance Service Publishing.

Spence, S.H. (1980). Social Skills Training with Children and Adolescents: A Counsellor’s Manual. Windsor: NFER-Nelson.

Spence, S. H. (1995). *Social skills training: Enhancing social competence with children and adolescents*. Nfer-Nelson.

Spreng, R. N., & Grady, C. L. (2010). Patterns of brain activity supporting autobiographical memory, prospection, and theory of mind, and their relationship to the default mode network. *Journal of cognitive neuroscience*, *22*(6), 1112-1123.

Swanson, J. M., Schuck, S., Porter, M. M., Carlson, C., Hartman, C. A., Sergeant, J. A., ... & Wigal, T. (2012). Categorical and dimensional definitions and evaluations of symptoms of ADHD: history of the SNAP and the SWAN rating scales. *The International journal of educational and psychological assessment*, *10*(1), 51.

Swettenham, J. (1996). Can children with autism be taught to understand false belief using computers?. *Journal of Child Psychology and psychiatry*, *37*(2), 157-165.

Tanaka, J. W., Wolf, J. M., Klaiman, C., Koenig, K., Cockburn, J., Herlihy, L., ... & Schultz, R. T. (2010). Using computerized games to teach face recognition skills to children with autism spectrum disorder: the Let’s Face It! program. *Journal of Child Psychology and Psychiatry*, *51*(8), 944-952.

Thapar, A., Cooper, M., & Rutter, M. (2017). Neurodevelopmental disorders. The Lancet Psychiatry, 4(4), 339-346.

Traverso, L., Viterbori, P., & Usai, M. C. (2015). Improving executive function in childhood: evaluation of a training intervention for 5-year-old children. *Frontiers in psychology*, *6*, 525.

Tseng, W. L., & Gau, S. S. F. (2013). Executive function as a mediator in the link between attention‐deficit/hyperactivity disorder and social problems. *Journal of Child Psychology and Psychiatry*, *54*(9), 996-1004.

Vahabzadeh, A., Keshav, N. U., Salisbury, J. P., & Sahin, N. T. (2018). Improvement of attention-deficit/hyperactivity disorder symptoms in school-aged children, adolescents, and young adults with autism via a digital smartglasses-based socioemotional coaching aid: short-term, uncontrolled pilot study. *JMIR mental health*, *5*(2), e9631.

Vajawat, B., Varshney, P., & Banerjee, D. (2021). Digital gaming interventions in psychiatry: evidence, applications and challenges. *Psychiatry research*, *295*, 113585.

Valentine, A. Z., Brown, B. J., Groom, M. J., Young, E., Hollis, C., & Hall, C. L. (2020). A systematic review evaluating the implementation of technologies to assess, monitor and treat neurodevelopmental disorders: A map of the current evidence. *Clinical psychology review*, *80*, 101870.

Vaughan, N., Gabrys, B., & Dubey, V. N. (2016). An overview of self-adaptive technologies within virtual reality training. *Computer Science Review*, *22*, 65-87.

Wainer, A. L., & Ingersoll, B. R. (2011). The use of innovative computer technology for teaching social communication to individuals with autism spectrum disorders. *Research in Autism Spectrum Disorders*, *5*(1), 96-107.

Wang, M., & Reid, D. (2011). Virtual reality in pediatric neurorehabilitation: attention deficit hyperactivity disorder, autism and cerebral palsy. *Neuroepidemiology*, *36*(1), 2-18.

Wang, M., & Reid, D. (2013). Using the virtual reality-cognitive rehabilitation approach to improve contextual processing in children with autism. *The Scientific World Journal*, *2013*.

Wang, X., Laffey, J., Xing, W., Ma, Y., & Stichter, J. (2016). Exploring embodied social presence of youth with Autism in 3D collaborative virtual learning environment: A case study. *Computers in Human Behavior*, *55*, 310-321.

Wass, S. V., & Porayska-Pomsta, K. (2014). The uses of cognitive training technologies in the treatment of autism spectrum disorders. *Autism*, *18*(8), 851-871.

Wechsler, D. (1991). *Wechsler intelligence scale for children* (3rd ed.). San Antonio, TX: The

Psychological Corporation.

Wechsler, D. (1997). Wechsler Adult Intelligence Scale (3rd edn). San Antonio, TX: Psychological Corporation.

Wechsler, D. Wechsler Abbreviated Scale of Intelligence. San Antonio, TX: The Psychological Corporation; 1999.

Wechlser, D. Wechsler Intelligence Scale for Children (WISC-IV). 4. San Antonio, TX: The Psychological Corporation; 2003.

Wechsler D (2004) The Wechsler Intelligence Scale for Children. 4th ed. London: Pearson Assessment.

Weerdmeester, J., Cima, M., Granic, I., Hashemian, Y., Gotsis, M., 2016. A Feasibility Study on the Effectiveness of a Full-Body Videogame Intervention for Decreasing Attention Deficit Hyperactivity Disorder Symptoms. Games Health J 5, 258–269.

Wellman, H. M., & Liu, D. (2004). Scaling of theory‐of‐mind tasks. *Child development*, *75*(2), 523-541.

Wieckowski, A. T., & White, S. W. (2017). Application of technology to social communication impairment in childhood and adolescence. *Neuroscience & Biobehavioral Reviews*, *74*, 98-114.

Williams, J., Scott, F., Stott, C., Allison, C., Bolton, P., Baron-Cohen, S., & Brayne, C. (2005). The CAST (childhood asperger syndrome test) test accuracy. Autism, 9(1), 45-68.

Williams, B. T., Gray, K. M., & Tonge, B. J. (2012). Teaching emotion recognition skills to young children with autism: a randomised controlled trial of an emotion training programme. *Journal of Child Psychology and Psychiatry*, *53*(12), 1268-1276.

Wilson, B. A., Alderman, N., Burgess, P. W., Emslie, H. C., & Evans, J. J. (1986). Behavioral Assessment of the Dysexecu- tive Syndome. Thames Valley Test Company: Flempton, Bury St. Edmunds.

Winner, M. G., & Crooke, P. (2014). Executive functioning and social pragmatic communication skills: Exploring the threads in our social fabric. *Perspectives on Language Learning and Education*, *21*(2), 42-50.

Whyte, E. M., Smyth, J. M., & Scherf, K. S. (2015). Designing serious game interventions for individuals with autism. *Journal of autism and developmental disorders*, *45*(12), 3820-3831.

Yan, N., Wang, J., Liu, M., Zong, L., Jiao, Y., Yue, J., ... & Liu, Z. (2008). Designing a brain-computer interface device for neurofeedback using virtual environments. *Journal of Medical and Biological Engineering*, *28*(3), 167-172.

Yeh, S. C., Tsai, C. F., Fan, Y. C., Liu, P. C., & Rizzo, A. (2012, December). An innovative ADHD assessment system using virtual reality. In *2012 IEEE-EMBS Conference on Biomedical Engineering and Sciences* (pp. 78-83). IEEE.

Yuan, S. N. V., & Ip, H. H. S. (2018). Using virtual reality to train emotional and social skills in children with autism spectrum disorder. *London journal of primary care*, *10*(4), 110-112.

Zelazo, P. D. (2015). Executive function: Reflection, iterative reprocessing, complexity, and the developing brain. *Developmental Review*, *38*, 55-68.

Zhu, H., Sun, Y., Zeng, J., & Sun, H. (2011). Mirror neural training induced by virtual reality in brain–computer interfaces may provide a promising approach for the autism therapy. *Medical hypotheses*, *76*(5), 646-647.
